# Antisense-mediated exon skipping: a therapeutic strategy for titin-based dilated cardiomyopathy

**DOI:** 10.15252/emmm.201505047

**Published:** 2015-03-10

**Authors:** Michael Gramlich, Luna Simona Pane, Qifeng Zhou, Zhifen Chen, Marta Murgia, Sonja Schötterl, Alexander Goedel, Katja Metzger, Thomas Brade, Elvira Parrotta, Martin Schaller, Brenda Gerull, Ludwig Thierfelder, Annemieke Aartsma-Rus, Siegfried Labeit, John J Atherton, Julie McGaughran, Richard P Harvey, Daniel Sinnecker, Matthias Mann, Karl-Ludwig Laugwitz, Meinrad Paul Gawaz, Alessandra Moretti

**Affiliations:** 1 Department of Cardiology and Cardiovascular Diseases, Eberhard Karls UniversityTübingen, Germany; 2 Victor Chang Cardiac Research InstituteDarlinghurst, NSW, Australia; 3 I. Medical Department – Cardiology, Klinikum rechts der Isar – Technische Universität MünchenMunich, Germany; 4 Department of Proteomics and Signal Transduction, Max Planck Institute of BiochemistryMartinsried, Germany; 5 Department of Biomedical Sciences, University of PadovaPadua, Italy; 6 Department of Experimental and Clinical Medicine, University Magna Graecia of CatanzaroCatanzaro, Italy; 7 Department of Dermatology, Eberhard Karls UniversityTübingen, Germany; 8 Libin Cardiovascular Institute of Alberta and University of CalgaryCalgary, AB, Canada; 9 Max Delbrueck Center for Molecular MedicineBerlin, Germany; 10 Department of Human Genetics, Leiden University Medical CenterLeiden, The Netherlands; 11 Institute for Integrative Pathophysiology, Universitätsmedizin MannheimMannheim, Germany; 12 Department of Cardiology, Royal Brisbane and Women's Hospital and University of Queensland School of MedicineBrisbane, Australia; 13 Genetic Health Queensland, Royal Brisbane and Women's HospitalBrisbane, Qld, Australia; 14 St Vincent's Clinical School, University of New South WalesKensington, NSW, Australia; 15 DZHK (German Centre for Cardiovascular Research) – partner site Munich Heart AllianceMunich, Germany

**Keywords:** dilated cardiomyopathy, exon skipping, induced pluripotent stem cells, titin

## Abstract

Frameshift mutations in the *TTN* gene encoding titin are a major cause for inherited forms of dilated cardiomyopathy (DCM), a heart disease characterized by ventricular dilatation, systolic dysfunction, and progressive heart failure. To date, there are no specific treatment options for DCM patients but heart transplantation. Here, we show the beneficial potential of reframing titin transcripts by antisense oligonucleotide (AON)-mediated exon skipping in human and murine models of DCM carrying a previously identified autosomal-dominant frameshift mutation in *titin* exon 326. Correction of *TTN* reading frame in patient-specific cardiomyocytes derived from induced pluripotent stem cells rescued defective myofibril assembly and stability and normalized the sarcomeric protein expression. AON treatment in *Ttn* knock-in mice improved sarcomere formation and contractile performance in homozygous embryos and prevented the development of the DCM phenotype in heterozygous animals. These results demonstrate that disruption of the *titin* reading frame due to a truncating DCM mutation can be restored by exon skipping in both patient cardiomyocytes *in vitro* and mouse heart *in vivo*, indicating RNA-based strategies as a potential treatment option for DCM.

## Introduction

Dilated cardiomyopathy (DCM) is a genetically heterogeneous disorder, with mutations in genes encoding cytoskeletal, nucleoskeletal, mitochondrial, and calcium-handling proteins (Hershberger & Siegfried, 2011). Most recently, however, approximately 25% of familial and 18% of sporadic DCM cases have been associated with truncating mutations in the *TTN* gene encoding titin (Herman *et al*, 2012), an extensively modular sarcomeric protein of more than 35,000 amino acids containing many repeating fibronectin-like and Ig-like domains (LeWinter *et al*, 2007; Chauveau *et al*, 2014). Titin is the biggest known single-copy protein in humans and spans the length of half the sarcomere, where it acts as a stretch sensor transmitting signals from its anchor at the Z-disk to its carboxyterminal kinase (TK) domain at the M-band (Kruger & Linke, 2009; Gautel, 2011). Previous work from our group identified, in a large Australian family, the first human mutation in *TTN* as a molecular basis for DCM. The 2-bp insertion, located in exon 326 (c.43628insAT, p.Ser14450fsX4), causes a frameshift with a premature stop codon in A-band titin (Gerull *et al*, 2002). Subsequent knock-in of this mutation in the mouse *Ttn* gene demonstrated embryonic lethality of homozygous animals due to severe defects in sarcomere assembly, while heterozygous mice are viable and develop DCM when exposed to cardiac stress (Gramlich *et al*, 2009).

RNA-based therapeutics and splice-switching approaches have been explored over the past decade for treating various diseases, including Duchenne muscular dystrophy (DMD), a severe muscle disorder caused by mutations in the large modular protein dystrophin (Aartsma-Rus, 2010). Antisense oligonucleotide (AON)-mediated exon skipping aimed at reframing dystrophin transcripts has been translated into clinical trials (van Deutekom *et al*, 2007; Kinali *et al*, 2009; Cirak *et al*, 2011; Goemans *et al*, 2011). Recently, an exon skipping strategy has been exploited in a *Mybpc3*-targeted knock-in mouse model of hypertrophic cardiomyopathy to enhance the expression of a naturally spliced functional mRNA variant (Gedicke-Hornung *et al*, 2013).

**Figure 1:**
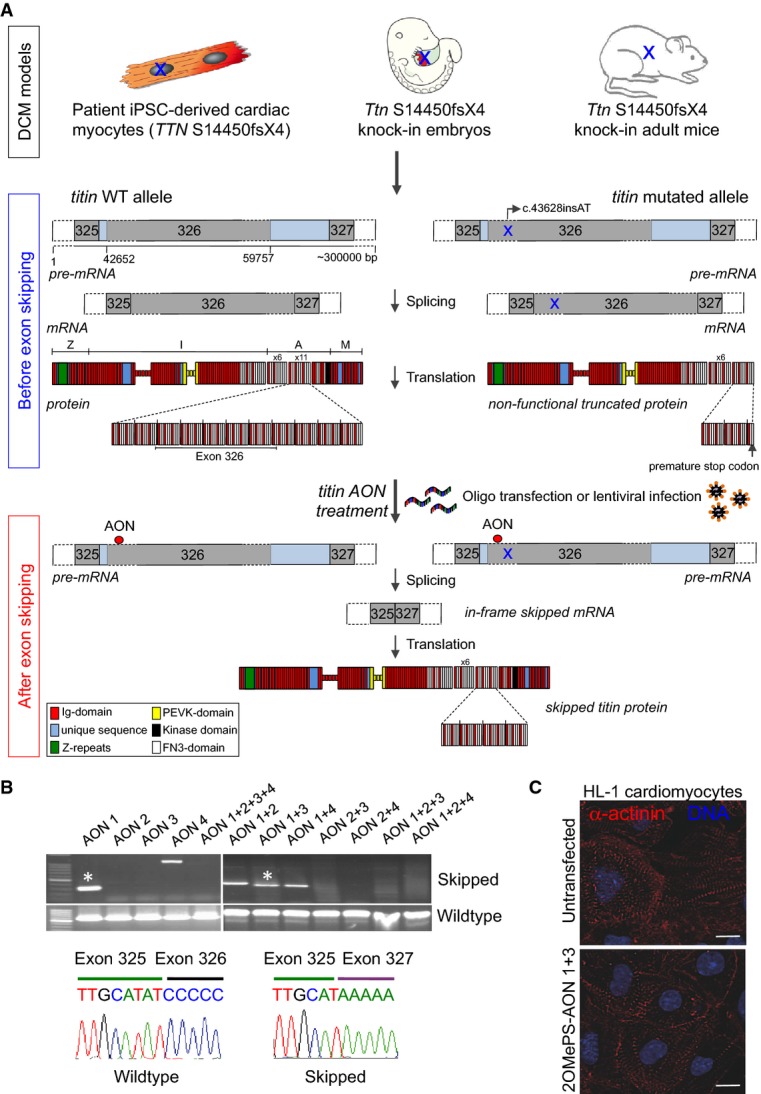
Exon skipping strategy and evaluation of 2OMePS antisense oligonucleotides (AONs) in HL-1 mouse cardiomyocytes

Here, we used patient-specific induced pluripotent stem cells (iPSCs) derived from an affected member of the Australian DCM family and the corresponding mouse knock-in DCM model (Gramlich *et al*, 2009) to investigate the potential of AON-mediated exon skipping as a therapeutic strategy to restore *titin* reading frame (Fig1A) and preserve myocardial function in DCM.

Schematic of the study design.

RT–PCR analysis (top) and representative direct sequencing (bottom) of *Ttn* exon 326 transcripts from HL-1 cardiomyocytes transiently transfected with different 2OMePS AONs. Only 2OMePS-AON1 and 2OMePS-AON1 + 3 (*) lead to a correct excision of exon 326 as confirmed by direct sequencing.

Immunofluorescence images of sarcomeric α-actinin in untransfected and 2OMePS-AON1 + 3-transfected HL-1 cardiomyocytes. Scale bars, 10 μm.

## Results

### AON-mediated skipping of *Ttn* exon 326 in HL-1 cardiomyocytes

We designed four different AON sequences that mask exonic splicing enhancer (ESE) motifs in *Ttn* exon 326 (Supplementary Table S1), whose stability was enhanced by phosphorothioate intersubunit linkages and a 2'-*O* modification of the ribose (2'-*O*-methyl phosphorothioate, 2OMePS), and first evaluated their efficacy in the mouse atrial cardiomyocyte tumor cell line HL-1 (Fig1B and C and Supplementary Fig S1). RT–PCR analysis (see Supplementary Fig S2 for PCR strategy) and direct sequencing demonstrated that only 2OMePS-AON1 and the combination of 2OMePS-AON1 and 3 were able to specifically block integration of exon 326 into the transcript with maintenance of the reading frame. 2OMePS-AON 2, 3, 4, and other combinations with 2OMePS-AON1 led to no or incorrect skipping of the exon, suggesting that the different AONs can interfere with each other (Fig1B). Moreover, immunofluorescence analysis for the Z-disk protein α-actinin in 2OMePS-AON1 + 3-transfected HL-1 cells showed a preserved sarcomere structure (Fig1C), indicating that loss of *Ttn* exon 326 can be accommodated by the cardiomyocytes.

### AON-mediated skipping of *TTN* exon 326 in patient-specific cardiomyocytes carrying the *TTN* Ser14450fsX4 mutation

**Figure 2:**
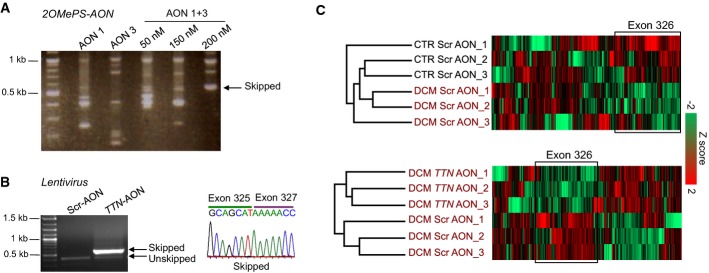
AON-mediated skipping of exon 326 in *TTN* Ser14450fsX4 induced pluripotent stem cell (iPSC)-derived cardiomyocytes

We further validated the efficiency of AON1 and AON3 sequences in skipping the human mutated *TTN* exon 326 by generating virus-free iPSCs from a 62-year-old female affected member of the DCM family carrying the heterozygous *TTN* Ser14450fsX4 mutation. The presence of the 2-bp AT insertion in exon 326 was confirmed by genomic sequencing (Supplementary Fig S3A). After characterization (Supplementary Figs S3 and S4), two iPSC clones from the DCM patient and an unrelated healthy female were chosen for further studies. Transient transfection of iPSC-derived cardiomyocytes with different doses and combinations of 2OMePS-AONs targeting the human *TTN* exon 326 and corresponding to the mouse AON1 and AON3 (Supplementary Table S1) resulted in incomplete and unspecific skipping of exon 326 at all concentrations tested, although 2OMePS-AON1 + AON3 promoted the highest amount of correctly skipped transcript in a dose-dependent manner (Fig2A). This was likely due to low transduction efficiency, as demonstrated by transfection of a 5′-fluorescein-labeled 2OMePS AON (Supplementary Fig S5A). In order to enhance nuclear AON delivery in human primary cardiomyocytes, we engineered a lentiviral construct in which the human AON1 and AON3 sequences were embedded in a modified U7 small-nuclear RNA (U7snRNA) followed by an IRES-GFP cassette (U7snRNA-*TTN*AONs-IRES-GFP). A lentivirus encoding mismatched scrambled AON sequences (U7snRNA-ScrAONs-IRES-GFP) was used as control (Supplementary Fig S5B). U7snRNA is normally involved in histone pre-mRNA 3'-end processing (Galli *et al*, 1983) and by a small change in the Sm/Lsm protein-binding sites (Stefanovic *et al*, 1995) can be directed to the spliceosome and used as a shuttle for antisense sequences (Goyenvalle, 2012). After lentiviral infection, ~85% of both control and DCM iPSC-derived cardiomyocytes were GFP positive (GFP^+^) (Supplementary Fig S5C), and a virtually complete, specific skipping of *TTN* exon 326 was achieved in both groups exclusively with U7snRNA-*TTN*AONs-IRES-GFP, as detected by RT–PCR and sequencing (Fig2B and Supplementary Fig S6A). We further confirmed the efficiency of *TTN* exon 326 skipping at the protein level using mass spectrometry (MS)-based shotgun proteomics (Fig2C and Supplementary Fig S6B). Among ~63,000 detected total peptides, 1,719 corresponded to the human titin protein and 298 mapped to the part encoded by exon 326. Unsupervised hierarchical clustering of titin peptides from control and DCM cardiomyocytes infected with U7snRNA-ScrAONs-IRES-GFP highlighted the presence of a cluster, which was significantly enriched in exon 326 peptides and down-regulated in the diseased cells, as expected (Fig2C). Down-regulation of exon 326 was also detected in DCM cells after treatment with U7snRNA-*TTN*AONs-IRES-GFP when compared to Scr-AONs, confirming specific skipping of the targeted exon (Fig2C). Similar results were obtained in control cells (Supplementary Fig S7B).

RT–PCR analysis of *TTN* exon 326 transcripts from DCM cardiomyocytes transiently transfected with 2OMePS-AON1, 2OMePS-AON3, and 2OMePS-AON1 + 3.

RT–PCR analysis (left) and representative direct sequencing (right) of *TTN* exon 326 transcripts from DCM cardiomyocytes infected with the U7snRNA-*TTN*AONs-IRES-GFP lentiviral vector carrying the AON1 and 3 sequences (*TTN*-AON) or with a control vector (U7snRNA-ScrAONs-IRES-GFP).

Mass spectrometry-based analysis of titin peptides in cells infected with the U7snRNA-ScrAONs-IRES-GFP (Scr-AON) and U7snRNA-*TTN*AONs-IRES-GFP (*TTN*-AON) vectors. Unsupervised hierarchical clustering identified a cluster significantly enriched in peptides mapping to exon 326 that was down-regulated in DCM Scr-AON cardiomyocytes compared to CTR Scr-AON cardiomyocytes (*n *=* *3, *P *=* *9.03E−8, Fisher's exact test, FDR = 0.04, top). Down-regulation of exon 326 was also detected in DCM *TTN*-AON cells when compared to DCM Scr-AON cells (*n *=* *3, *P *=* *0.02, Fisher's exact test, bottom).

### Rescue of sarcomeric assembly and stability in *TTN* Ser14450fsX4 iPSC-derived cardiomyocytes by skipping of *TTN* exon 326

**Figure 3:**
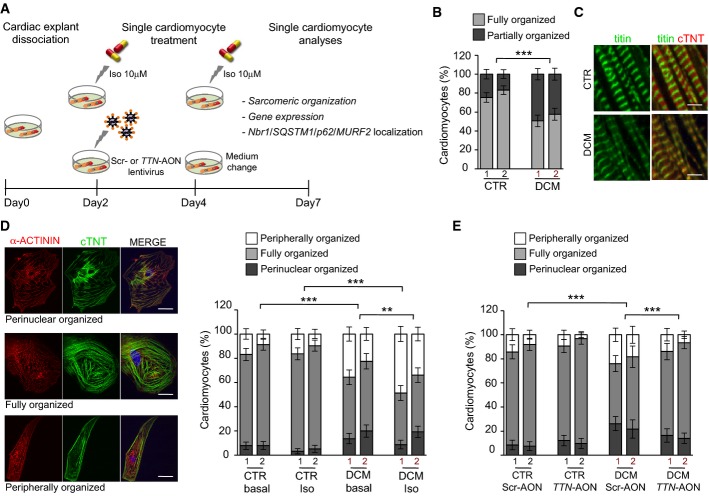
Exon skipping-based rescue of sarcomeric defects in *TTN* Ser14450fsX4 iPSC-derived cardiomyocytes

We next investigated the impact of the *TTN* A-band truncating mutation on sarcomere organization of iPSC-derived cardiomyocytes and evaluated the effects of reframing *TTN* transcripts by exon skipping in patient-specific DCM cells (Fig3A). We dissociated single cardiomyocytes from spontaneously beating foci and analyzed them 7 days later, to provide the time necessary for reorganization of the myofibrils that typically undergo disarray during the dissociation process (Atherton *et al*, 1986). Initial immunocytochemical analysis for titin and various proteins marking different portions of the sarcomere—α-actinin (Z-disk), cardiac troponin T (cTNT, A-band), and myosin heavy chain (MHC, M-line)—revealed, in the DCM group, a higher percentage of cardiomyocytes in which organized myofibrils occupied only half of the whole cytoplasm or less (Fig3B). Moreover, the immunofluorescence signal of Z-disk titin in striated myofibrils appeared more diffuse in patient cells (Fig3C). These results suggested that truncated titin mutants could alter assembly and/or stability of the sarcomeric units in human embryonic myocytes. To gain a better understanding of sarcomere remodeling in iPSC-derived cardiomyocytes after dissociation, we stably overexpressed actin as a fusion with a red fluorescent protein (RFP) in single cells by baculovirus technology (Supplementary Fig S7A), which has been recently successfully applied to mammalian cells (Sung *et al*, 2014), and performed live-cell imaging over time (Supplementary Fig S7B). This analysis showed that, similarly to neonatal cardiomyocytes (Atherton *et al*, 1986), there is a considerable heterogeneity in the degree of myofibril organization at day 1–2 after dissociation, but most of the cells presented striated RFP^+^ sarcomeres mainly around the nucleus, suggesting this as starting site of myofibril reassembly. With the time, the myofibril organization extended to the whole cell and this associated with the onset of rhythmic contraction. However, around 7–10 days of culture as single cells, few of the ‘fully organized' cardiomyocytes began to lose their sarcomeric striated pattern, starting at the perinuclear region and eventually throughout the whole cytoplasm till the cell periphery (Supplementary Fig S7B). On the basis of these observations, we hypothesized that sarcomere remodeling in iPSC-derived cardiomyocytes after dissociation is a radially occurring process that mimics the one earlier observed in neonatal cardiomyocyte (Atherton *et al*, 1986).

Experimental design.

Percentage of iPSC-derived cardiomyocytes with cross-striated myofibrils occupying a fraction (partially organized, dark gray) or the whole cytoplasm (fully organized, light gray) from two control and two patient clones. Statistical difference was tested using the two-sided chi-squared test (CTR1 basal: *n *=* *283, CTR2 basal: *n *=* *250, DCM1 basal: *n *=* *255, DCM2 basal: *n *=* *236; ***P =* *1.93E−16).

Immunofluorescence images of titin (Z-disk portion) and cardiac troponin T (cTNT, A-band) in well-organized sarcomeres from CTR and DCM single cardiomyocytes under basal conditions. Scale bars, 5 μm.

Immunofluorescence images (left) of α-actinin and cTNT in CTR and DCM single cardiomyocytes, illustrating 3 different levels of sarcomeric organization (perinuclear, fully and peripherally organized). Percentage of cells with different levels of sarcomeric organization (right) under basal and stress conditions. Statistical difference was tested using the chi-squared test (CTR1 basal: *n *=* *283, CTR2 basal: *n *=* *250, DCM1 basal: *n *=* *255, DCM2 basal: *n *=* *236, CTR1 Iso: *n *=* *245, CTR2 Iso: *n *=* *230; DCM1 Iso: *n *=* *242 and DCM2 Iso: *n *=* *269; ***P =* *1.93E−16 CTR basal versus DCM basal; ***P =* *9.61E−34 CTR Iso versus DCM Iso, **P =* *0.001 DCM basal versus DCM Iso). No significant differences were observed comparing CTR basal and CTR Iso groups. Scale bars, 25 μm.

Percentage of perinuclear, fully, and peripherally organized single cardiomyocytes from two CTR and two DCM iPSC clones after infection with the U7snRNA-ScrAONs-IRES-GFP and U7snRNA-*TTN*AONs-IRES-GFP lentiviruses. Statistical difference was tested using the two-sided chi-squared test (CTR1 Scr-AON: *n *=* *190, CTR2 Scr-AON: *n *=* *200, DCM1 Scr-AON: *n *=* *221, DCM2 Scr-AON: *n *=* *115, CTR1 *TTN*-AON: *n *=* *223, CTR2 *TTN*-AON: *n *=* *187, DCM1 *TTN*-AON: *n *=* *171, DCM2 *TTN*-AON: *n *=* *243; ***P =* *4.22E−15 CTR Scr-AON versus DCM Scr-AON; ***P =* *4.61E−02 DCM Scr-AON versus DCM *TTN*-AON). No significant differences were observed comparing CTR Scr-AON and CTR *TTN*-AON groups.

A subsequent detailed analysis of cellular myofilament arrangement in iPSC-derived cardiomyocytes 7 days after dissociation demonstrated that ~80% of control and only 50% of DCM cells had structured myofibrils occupying the entire cytoplasm under basal conditions (Fig3D). On the contrary, the fractions of cells with organized sarcomeres only in the perinuclear or peripheral region were significantly increased in the patient group (Fig3D), suggesting defects in both myofibril reassembly and sarcomere stability. Moreover, isoproterenol (Iso) treatment exacerbated the phenotype of DCM cardiomyocytes, with no significant impact on the control counterparts (Fig3D), demonstrating that patient cells are more susceptible to catecholamine-induced stress. Importantly, infection with the U7snRNA-*TTN*AONs-IRES-GFP lentivirus followed by analysis of striated myofibril distribution in GFP^+^ cardiomyocytes revealed that skipping of *TTN* exon 326 partially rescued the sarcomere abnormalities of DCM cells, while no effects were observed in the control group or after infection with the U7snRNA-ScrAONs-IRES-GFP virus (Fig3E). Lentiviral infection had no influence on sarcomere remodeling in both healthy and disease cellular settings (Supplementary Fig S8).

### Normalization of sarcomeric protein expression and Nbr1/p62/SQSTM1/MURF2 signalosome in *TTN* Ser14450fsX4 iPSC-derived cardiomyocytes by skipping of *TTN* exon 326

**Figure 4:**
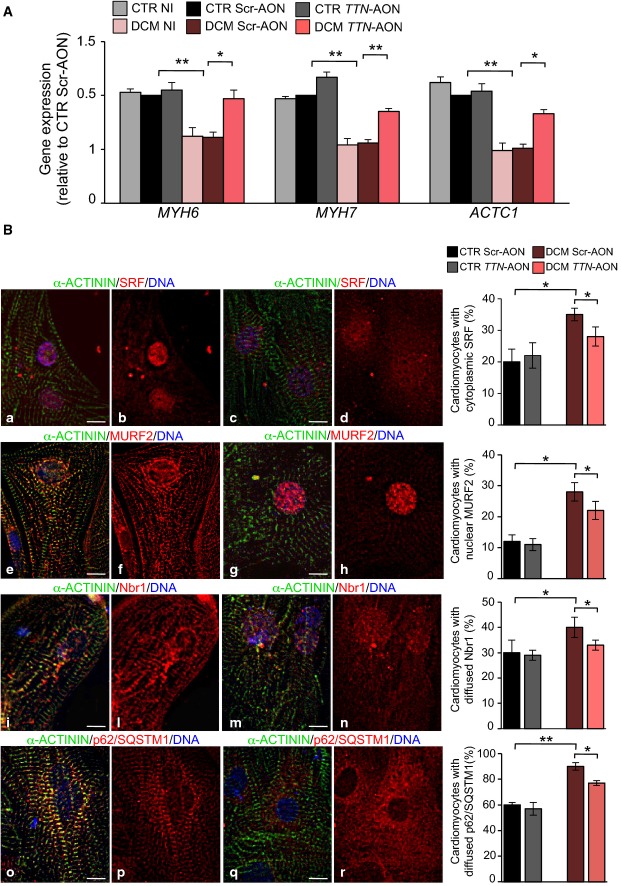
Exon skipping-based rescue of SRF target gene expression and Nbr1/p62/SQSTM1/MURF2 subcellular distribution in *TTN* Ser14450fsX4 iPSC-derived cardiomyocytes

Titin is not only required as molecular scaffold during sarcomerogenesis and assists in the process of myofibrillar assembly, but it is also a hot spot for protein–protein interactions and a putative mediator of mechanotransduction. About 20 interaction partners have so far been identified, linking titin to multiple stress signaling pathways that control muscle gene expression and protein turnover (Linke & Kruger, 2010; Linke & Hamdani, 2014). One of the most extensively studied is the Nbr1/p62/SQSTM1/MURF2 signaling complex that associates with titin TK and activates the serum response factor (SRF) upon mechanical stimuli (Lange *et al*, 2005). TK mutations affecting this interaction result in the dissociation of the Nbr1/p62/SQSTM1/MURF2 complex and translocation of MURF2 into the nucleus, which in turn leads to suppression of SRF-dependent muscle gene transcription (Lange *et al*, 2005). Therefore, we analyzed expression levels of SRF targets (Miano *et al*, 2004; Balza & Misra, 2006) in control and patient cardiomyocytes after infection with U7snRNA-ScrAONs-IRES-GFP and U7snRNA-*TTN*AONs-IRES-GFP viruses. When compared to the control counterpart, a significant down-regulation of α- and β-myosin heavy chain (*MYH6* and *MYH7*) transcripts as well as cardiac α-actin (*ACTC1*) was measured in the DCM cells untreated or transduced with scrambled AONs (Fig4A). Blocking of exon 326 transcription partially rescued SRF target levels in patient cardiomyocytes, with no effects on control cells (Fig4A). In concordance, immunocytochemistry assessment of SRF localization in iPSC-derived cardiomyocytes at 7 days after dissociation revealed a significant higher percentage of cells with increased extranuclear expression of SRF in the DCM group (Fig4B). Moreover, we observed differences in the subcellular distribution of MURF2, Nbr1, and p62/SQSTM1 in the DCM cardiomyocytes, with an increased number of cells showing a marked nuclear accumulation of MURF2 and a cytosolic, more diffused non-sarcomeric expression of Nbr1 and p62/SQSTM1 (Fig4B). Infection with U7snRNA-*TTN*AONs-IRES-GFP lentivirus partially normalized the cellular localization of all these proteins in the diseased cells, while no effects were observed in the control cells (Fig4B).

qRT–PCR analysis of SRF target genes (*MYH6*, *MYH7* and *ACTC1)* in CTR and DCM single cardiomyocytes under basal condition (no infection, NI) and after infection with control U7snRNA-ScrAONs-IRES-GFP (Scr-AON) and the U7snRNA-*TTN*AONs-IRES-GFP (*TTN*-AON) lentiviruses. Statistical difference was tested using the two-sided Student's *t*-test (**P =* *0.009, CTR Scr-AON versus DCM Scr-AON; *P =* *0.04, DCM Scr-AON versus DCM *TTN*-AON for *MYH6*; **P =* *0.002, CTR Scr-AON versus DCM Scr-AON; **P =* *0.002, DCM Scr-AON versus DCM *TTN*-AON for *MYH7*; **P =* *0.004, CTR Scr-AON versus DCM Scr-AON; *P =* *0.02, DCM Scr-AON versus DCM *TTN*-AON for *ACTC1*). No significant differences were observed comparing the CTR NI, CTR Scr-AON and CTR *TTN*-AON groups and comparing the DCM NI and DCM Scr-AON groups. Expression values were relative to CTR Scr-AON, normalized to *GAPDH*, and presented as mean ± SEM, *n *=* *3.

Immunofluorescence images showing normal (a, b, e, f, i, l, o, p) and altered (c, d, g, h, m, n, q, r) intracellular distribution of SRF (a and b, nuclear; c and d, cytoplasmic), MURF2 (e and f, sarcomeric; g and h, nuclear), Nbr1 (i and l, sarcomeric; m and n, diffused), and SQSTM1/p62 (o and p, sarcomeric; q and r, diffused) in representative single cardiomyocytes (left). Sarcomeres are marked by α-actinin. On the right, percentage of CTR and DCM cardiomyocytes showing cytoplasmic expression of SRF, nuclear accumulation of MURF2, and diffused expression of Nbr1 and of SQSTM1/p62 after infection with control U7snRNA-ScrambleAONs-IRES-GFP (Scr-AON) and the U7snRNA-*TTN*AONs-IRES-GFP (*TTN*-AON) lentiviruses (right). Data represent mean values ± SEM from two control and two DCM clones. Statistical difference was tested using the two-sided chi-squared test (CTR Scr-AON: *n *=* *874, *n *=* *874, *n *=* *882 and *n *=* *890, CTR *TTN*-AON: *n *=* *880, *n *=* *990, *n *=* *878 and *n *=* *890, DCM Scr-AON: *n *=* *890, *n *=* *887, *n *=* *884 and *n = 886*, DCM *TTN*-AON: *n *=* *900, *n *=* *875, *n *=* *899 and *n *=* *891 for SRF, MURF2, Nbr1 and SQSTM1/p62, respectively; *P =* *0.01, CTR Scr-AON versus DCM Scr-AON; *P =* *0.04, DCM Scr-AON versus DCM *TTN*-AON for SRF; *P =* *0.02, CTR Scr-AON versus DCM Scr-AON; *P =* *0.04, DCM Scr-AON versus DCM *TTN*-AON for MURF2; *P =* *0.03, CTR Scr-AON versus DCM Scr-AON; *P =* *0.03, DCM Scr-AON versus DCM *TTN*-AON for Nbr1; **P =* *0.009, CTR Scr-AON versus DCM Scr-AON; *P =* *0.01, DCM Scr-AON versus DCM *TTN*-AON for SQSTM1/p62). No significant differences were observed comparing CTR Scr-AON and CTR *TTN*-AON groups. Scale bars, 50 μm.

Taken together, these findings suggest that defective sarcomere assembly and stability of DCM myocytes may partly derive from reduced expression of structural sarcomeric proteins resulting from disruption of the Nbr1/p62/SQSTM1/MURF2 signalosome.

### Restoration of sarcomere assembly and cardiac function in mouse *Ttn* Ser14450fsX4 knock-in DCM embryos by skipping of *Ttn* exon 326

**Figure 5:**
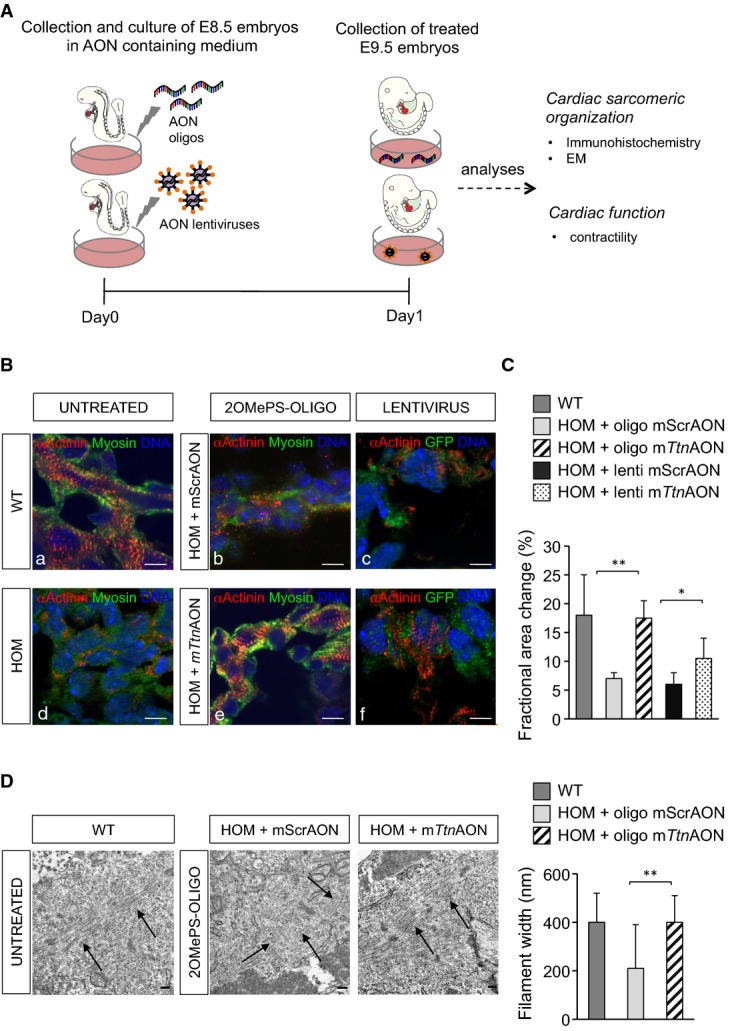
Exon skipping-based rescue of the DCM phenotype in *Ttn* Ser14450fsX4 knock-in embryos

We further assessed the efficacy and impact of reframing *Ttn* transcripts by AON-mediated skipping in the heart muscle and used the mouse DCM model based on the human *TTN* exon 326 A-band truncating mutation (Gramlich *et al*, 2009). We generated U7snRNA-AONs-IRES-GFP lentiviral constructs encoding the mouse specific AON1/3 (U7snRNA-m*Ttn*AONs-IRES-GFP) or scrambled AON (U7snRNA-mSrcAONs-IRES-GFP) sequences. We first analyzed knock-in mouse embryos, which were collected at day 8.5 and cultured for 24 h after lentiviral infection or transient transfection of mouse 2OMePS oligos—AON1/3 (m*Ttn*AON) or scrambled AONs (mScrAON)—in order to compare the efficacy of the two delivery methods (Fig5A). Wild-type (WT) untreated hearts displayed proper formation of sarcomeres with clearly distinguishable Z-disks (Fig5B) and beat normally (Fig5C). In contrast, homozygous *Ttn*-mutant myocardium of the same stage showed no striations (Fig5B), indicative of impaired sarcomerogenesis, and consequently did not develop contractile function (Fig5C). Mutants treated with m*Ttn*AONs exhibited rescued sarcomere assembly and Z-disk formation (Fig5B) and a significant increase in contractile function when compared to untreated or mScrAON-treated homozygous littermates (Fig5C). Moreover, m*Ttn*AON application restored normal filament width, as detected by electron microscopy (Fig5D and E). Beneficial effects after m*Ttn*AON treatment were similar in both 2OMePS- and lentivirus-based systems (Fig5B and C), suggesting a comparable efficacy of the two methods in transducing the myocardium *ex vivo*.

A Experimental design.

B, C Immunofluorescence images of sarcomeric α-actinin and myosin (B) and assessment of cardiac function (C) in homozygous *Ttn* knock-in embryos cultured for 24 h after mScrAON and m*Ttn*AON transient transfection (*n *=* *3 per group) or lentivirus delivery (*n *=* *7 per group). Scale bars, 10 μm.

D Electron microscopy analysis in *Ttn* knock-in embryos cultured for 24 h after mScrAON and m*Ttn*AON transient transfection. m*Ttn*AON treatment of homozygous embryos rescued myofibril formation (left, black arrows), resulting in thicker filaments (right). Data information: Statistical difference was tested using the two-sided Student's *t*-test (**P =* *0.007 and *P =* *0.01 for fractional area change and **P =* *0.009 for filament width differences). Data represent mean values ± SEM. Scale bars, 0.2 μm.

### Prevention of DCM development in heterozygous *Ttn* Ser14450fsX4 knock-in mice by skipping of *Ttn* exon 326

Next, we evaluated the effects of AON-mediated skipping of *Ttn* exon 326 on cardiac function in adult heterozygous *Ttn* knock-in mice (HET) *in vivo*. Since gene transfer using viral-based approaches has limited translational potential into the clinic, we sought to test whether efficient *Ttn* exon 326 skipping in the heart could be achieved by systemic administration of oligonucleotides.

**Figure 6:**
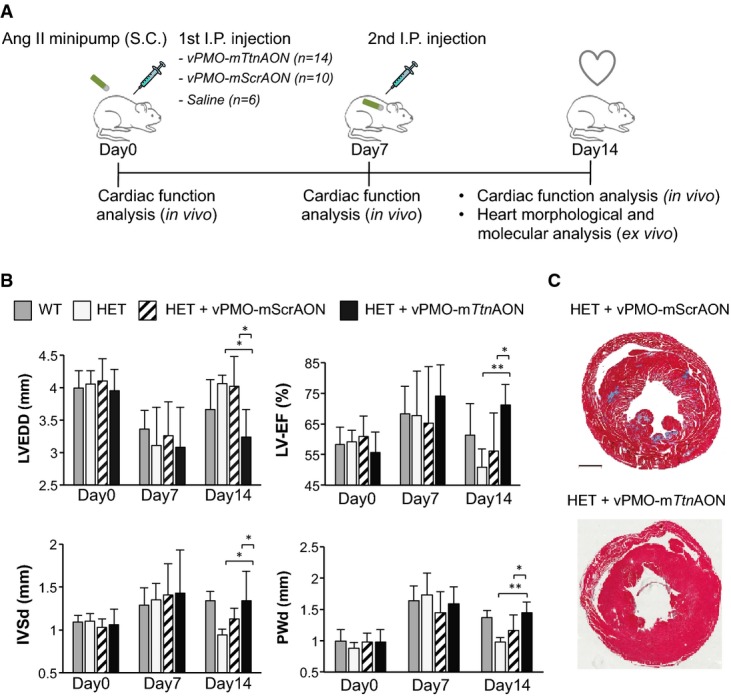
Prevention of DCM phenotype development in adult *Ttn* Ser14450fsX4 knock-in mice by injection of vivo-morpholino-modified antisense oligonucleotides

Although used in several of the preclinical and early clinical trials for DMD, the 2OMePS oligos are reported to have low effectiveness in targeting cardiac muscle *in vivo* (Heemskerk *et al*, 2009; Betts & Wood, 2013). Indeed, in a preliminary screening for AON chemistry that allows skipping of *Ttn* exon 326 in the heart *in vivo*, systemic infusion of the mouse 2OMePS-AON 1 and 3 did not produce any skipping of cardiac *Ttn* in HET animals, even at the highest doses tested (Supplementary Fig S9A), while local skeletal muscle injection of the same 2OMePS AONs led to correct skipping of such exon in the skeletal muscle transcript (Supplementary Fig S9A). Therefore, we chose to use vivo-morpholino-modified AONs (vPMO-AONs, see Materials and Methods), which have been described to better penetrate heart tissue (Mendell *et al*, 2013). Intraperitoneal (I.P.) injection of 6 mg/kg body weight of mouse vPMO-AON1 and 3 allowed skipping of cardiac *Ttn* exon 326 in HET mice, but in an incomplete and unspecific manner (Supplementary Fig S9B). However, extension of the v-PMO-AON1 sequence from a 23mer to a 28mer markedly improved AON efficiency and specificity (Supplementary Fig S9C). Therefore, the 28-mer vPMO-AON1 (vPMO-m*Ttn*AON) and its correspondent scrambled sequence (vPMO-mScrAON) were further used in our *in vivo* studies. Figure6A illustrates our experimental design. Heterozygous *Ttn* knock-in mice did not show any obvious cardiac phenotype under sedentary conditions. However, when exposed to cardiac stress they develop features of DCM (Gramlich *et al*, 2009). Thus, we implanted osmotic minipumps delivering Ang II (1.4 mg/kg) in WT (*n *=* *6) and HET animals (*n *=* *30). One subgroup of HET mice was intraperitoneally injected with 6 mg/kg body weight of vPMO-m*Ttn*AON (*n *=* *14) at day 0 and day 7. Another subgroup received the same dose of a mismatched vPMO-mScrAON (*n *=* *10) or no oligos (*n *=* *6) (Fig6A). Echocardiographic analysis of systolic and diastolic function revealed that all subgroups developed a similar hypertrophic response after 1 week of Ang II infusion, with decreased ventricular end-diastolic diameters (LVEDD) and increased left ventricular ejection fraction (LV-EF) and wall thicknesses (IVSd and PWd) (Fig6B). Consistent with our previous findings (Gramlich *et al*, 2009), after 2 weeks of Ang II HET animals that were injected with saline or vPMO-mScrAON displayed a DCM-like phenotype characterized by a reduction in LV-EF, IVSd, and PWd and by an increase in LVEDD (Fig6B). In contrast, vPMO-m*Ttn*AON-treated HET mutants did not develop DCM and showed a response similar to WT animals with continued hypertrophy (Fig6B). Furthermore, vPMO-m*Ttn*AON injections in HET animals reversed the development of interstitial fibrosis that typically occurs in these mice after a 2-week treatment with Ang II (Fig6C). At the end of each experiment, the animals were carefully analyzed for organ damage (e.g., liver failure). We could not detect any signs of toxicity in vPMO-treated mice.

Experimental design.

Echocardiographic analysis of adult knock-in mice. Statistical difference was tested using the two-sided Student's *t*-test (WT: *n *=* *6, HET: *n *=* *6; HET + vPMO-mScrAON: *n *=* *10 and HET + vPMO-m*Ttn*AON: *n *=* *14; *P =* *0.013, HET versus HET + vPMO-m*Ttn*AON and *P =* *0.02, HET + vPMO-mScrAON versus HET + vPMO-m*Ttn*AON for LVEDD differences; **P =* *0.006, HET versus HET + vPMO-m*Ttn*AON and *P =* *0.02, HET + vPMO-mScrAON versus HET + vPMO-m*Ttn*AON for LV-EF differences; *P =* *0.03, HET versus HET + vPMO-m*Ttn*AON and *P =* *0.04, HET + vPMO-mScrAON versus HET + vPMO-m*Ttn*AON for IVSd differences; **P =* *0.001, HET versus HET + vPMO-m*Ttn*AON and *P =* *0.03, HET + vPMO-mScrAON versus HET + vPMO-m*Ttn*AON and for PWd differences). No significant differences were observed among groups at day 0 and day 7. Data represent mean values ± SEM.

Masson's trichrome staining of heart sections from HET animals injected with vPMO-mScrAONs and vPMO-m*Ttn*AON. Scale bars, 1 mm.

**Figure 7:**
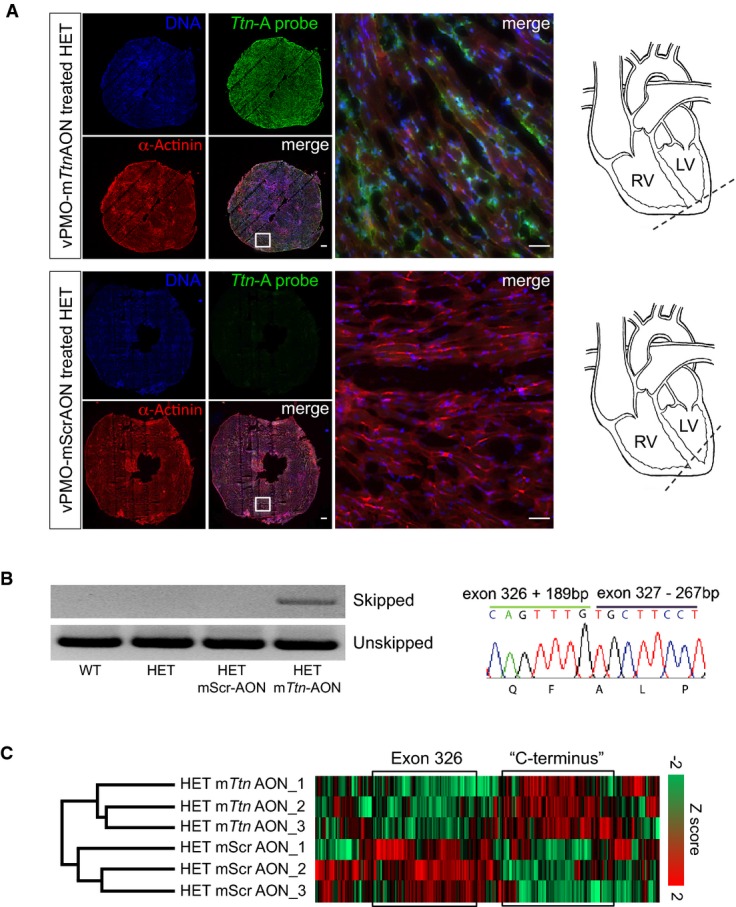
Efficient skipping of exon 326 *in vivo*-morpholino-modified antisense oligonucleotide-treated adult *Ttn* Ser14450fsX4 knock-in mice

To assess whether the functional improvement was associated with exon skipping, animals were sacrificed at the end of the 2-week treatment and molecular analyses were performed in ventricular tissue (Fig1). Fluorescence in situ hybridization (FISH) with a specific probe complementary to the m*Ttn*AON sequence (*Ttn*-A probe) demonstrated an almost complete penetrance of the vivo-morpholino into the heart of the vPMO-m*Ttn*AON-treated HET animals, while no signal was detected in HET mice that received the vPMO-mScrAON (Fig7A and Supplementary Fig S9D). RT–PCR and direct sequencing of cardiac cDNA demonstrated that vPMO-m*Ttn*AON treatment restored the *Ttn* reading frame, although the resulted transcript was shorter than expected, including the first 189 bp of exon 326 and lacking the first 267 bp of exon 327 (Fig7B). Finally, evaluation of titin protein by MS-based proteomic analysis revealed a down-regulation of peptides mapping to mouse exon 326 and an up-regulation of peptides downstream of exon 326 (that we refer as ‘C-terminus') in ventricular tissue of HET animals treated with vPMO-m*Ttn*AON compared to vPMO-mScrAON (Fig7C). By calculating the percentage change of the relative intensity of ‘C-terminus' peptides to all titin peptides, we estimated an ~8% skipping efficiency of the mutated 326 exon after the application of vPMO-m*Ttn*AON (see Supplementary Information for details). These results are in accordance with data in dystrophic *mdx* mice reporting a ~5–10% dystrophin induction in heart muscle after intravenous injection of 6–300 mg/kg morpholinoE23 (Wu *et al,* 2010).

Fluorescence *in situ* hybridization (FISH) of heart muscle tissue from adult knock-in mice with a probe complementary to vPMO-m*Ttn*AON. Scale bars, 250 and 50 μm (magnification).

RT–PCR analysis of *Ttn* exon 326 transcripts from heart tissue of untreated WT and HET animals and vPMO-mScrAON- and vPMO-m*Ttn*AON-treated mice (left). Representative direct sequencing of *Ttn* exon 326 transcripts from vPMO-m*Ttn*AON-treated HET heart tissue (right).

Mass spectrometry-based analysis of titin peptides in adult knock-in mice injected with vPMO-mScrAONs and vPMO-m*Ttn*AONs. Unsupervised hierarchical clustering identified a cluster enriched in exon 326 peptides that was down-regulated in vPMO-m*Ttn*AON-treated animals compared to vPMO-mScrAON-treated littermates. Another cluster enriched in C-terminal peptides was up-regulated in the vPMO-m*Ttn*AON group compared to the vPMO-mScrAON group (*n *=* *3, *P *=* *0.02, Fisher's exact test, FDR = 0.04).

Taken together, these results demonstrate an effective targeting of the myocardium by systemic application of vPMO-AONs and suggest that partial skipping of the mutated exon 326 with restoration of *Ttn* open reading frame is sufficient to prevent the development of the DCM phenotype *in vivo*.

## Discussion

Mutations in *TTN* are a major cause for DCM, occurring in approximately 30% of cases (Herman *et al*, 2012). The bulk of them is located in the A-band portion of the protein and mainly produces truncated products lacking part of the A region, which associates with the myosin filament, and the M-band, which encompasses the TK and several adjoining domains and is established as a hot spot for protein–protein interactions, including cytoskeletal and signaling proteins as well as protein turnover regulators (Herman *et al*, 2012; Chauveau *et al*, 2014; Linke & Hamdani, 2014).

Our study demonstrates that RNA-based rescue of the A-band truncating *titin* Ser14450fsX4 mutation in exon 326 ameliorates the DCM phenotype at both structural and functional levels in mouse and patient-specific models of the disease. We used the HL-1 cardiomyocyte cell line to evaluate optimal sequence, chemistry and combination of AONs to block integration of *Ttn* exon 326 into the transcript. We showed that excision of this exon has a negligible effect on sarcomere structure in both HL-1 cells and human healthy iPSC-derived cardiomyocytes. Skipping of the mutated exon in patient-specific cardiomyocytes carrying the *TTN* Ser14450fsX4 mutation improved myofibril assembly and stability and normalized the expression of muscle genes regulated by TK. Finally, we validated the skipping approach in *Ttn* knock-in mutant mice and were able to rescue the DCM phenotype of homozygous and heterozygous animals.

Little is known about the precise biological and pathophysiological mechanisms related to the different DCM *TTN* mutations, due to the limited availability of patients' myocardial tissue and the technical difficulties of studying such a huge and complex protein. In patient-specific iPSC-derived cardiomyocytes carrying the truncating A-band *TTN* Ser14450fsX4 mutation we observed defects in building up and maintenance of a stable sarcomeric structure (Fig3), which were associated with perturbations of the TK interacting Nbr1/p62/SQSTM1/MURF2 signalosome and reduction of SRF-dependent muscle gene expression (Fig4). Since the Nbr1/p62/SQSTM1/MURF2 complex regulates ubiquitin/proteasome and autophagy/lysosomal pathways as well as SRF transcriptional program (Lange *et al*, 2005; Kotter *et al*, 2014; Linke & Hamdani, 2014), both pathophysiological mechanisms are likely to be involved in the observed myofibril phenotype. Moreover, since the Ser14450fsX4 mutation leads to a truncated titin protein missing a more than half of the A-band and the entire M-band, and thereby lacking a strong connection with the thick filaments and many important protein–protein interactions, several other structural and signaling effects are expected to play a major role in the disease phenotype.

Exon 326 is the largest exon of titin and consists entirely of highly repetitive Ig and FN III motifs, which might explain why its loss by exon skipping can be tolerated by the organism. Out of the 69 reported *TTN* mutations associated with DCM, 14 of them (seven frameshift variants, six nonsense changes, and one predicted splicing mutation) are located in exon 326 (Chauveau *et al*, 2014). Thus, skipping of such exon may provide a molecular rescue of ~20% of the known DCM-causing *TTN* mutations. It is noteworthy that exon skipping events in *TTN* also occur naturally and modulate the fractional extensions of the tandem Ig and PEVK (region that is rich in proline, glutamate, valine, and lysine) segments, thereby influencing myofibrillar elasticity (Freiburg *et al*, 2000). Not all of the DCM-associated variants, however, may be amenable to exon skipping therapy in equal ways (e.g., missense, compound heterozygous, and splicing mutations as well as variants in asymmetric exons or exons with nonredundant domains). Thus, it would be of great interest to investigate whether the applicability of exon skipping could be extended to other *TTN* exons belonging to the A-band mutation hot spot region and to other types of DCM-causing non-truncating *TTN* mutations, which would broaden such a therapeutic approach to larger cohorts of DCM patients. Our work demonstrates that patient-specific iPSCs can provide an exciting new cellular platform for such screening (Grskovic *et al*, 2011).

AON-mediated exon skipping aimed at reframing transcripts is emerging as a promising therapeutic strategy since the encouraging results of the recent phase 2/3 clinical trials for treatment of DMD (van Deutekom *et al*, 2007; Kinali *et al*, 2009; Cirak *et al*, 2011; Goemans *et al*, 2011). By demonstrating that a human DCM-causing *TTN* mutation can be corrected to preserve cardiac function, our proof of concept study suggests exon skipping as a potential treatment approach for a substantial proportion of DCM patients. Moreover, beneficial effects of exon skipping *in vivo* were achieved by systemic application of vPMO-AONs, a chemistry that is currently under extensive investigation and is rapidly becoming safer and more efficient (Betts *et al*, 2012).

One limitation of the current work is that the DCM phenotype in the adult *Ttn-*mutant mice needs to be induced by a cardiac stressor, for example, angiotensin II infusion. Simultaneous injections of m*Ttn*AONs prevented the development of heart failure and led to the expected hypertrophic response seen in WT animals. However, it remains unclear whether AON therapy is able to reverse an already existing DCM phenotype. This is the subject of ongoing investigation.

Current approaches to the management of DCM are focused on correcting deranged secondary pathophysiological processes, including neurohumoral activation and both structural and electrical cardiac remodeling. However, these treatments are generally applied late in the disease trajectory, with these patients remaining at high risk of death and hospitalization. In our work, we provide a novel RNA-based approach, which aims to address the underlying molecular pathophysiology of a sizable proportion of patients affected by DCM. This could potentially allow more effective management and, if applied early, the prevention of the heart failure phenotype. Future preclinical studies in large animal models will be needed to address optimal chemistry, application regime, and pharmaco-kinetics of AON *in vivo* for optimal rescue of DCM-associated truncating mutations in *TTN*.

## Materials and Methods

An extended Materials and Methods section is available in the Supplementary Information.

### Antisense oligonucleotide modifications

AONs were designed using previously published guidelines (Aartsma-Rus, 2012). 2OMePS-AONs consisted of 2'-*O*-methyl RNA, had a full-length phosphorothioate backbone, and were synthesized and purified at Eurogentec, Germany. In vPMOs (vivo-phosphorodiamidate morpholino oligomers), the sugar ribose backbone of the RNA was replaced by a six-membered morpholino moiety and a peptide conjugation with eight terminal guanidinium groups on a dendrimer scaffold was added. vPMO-AONs were synthesized and purified at GeneTools, LLC (Philomath, OR, USA) as described (Morcos *et al*, 2008).

### Individuals involved in the study

For the generation of iPSCs, we recruited a 62-year-old female affected member of the previously described DCM family (Gerull *et al*, 2002) and an unrelated age- and gender-matched control individual. The control had a normal health status without a history of cardiac disease or any cardiovascular risk factors.

This study was performed following a human research subject protocol approved by the Institutional Review Board and the Ethic Committee of the Klinikum rechts der Isar, Technische Universität München. Written informed consent was obtained from the affected patient and the healthy volunteer, and experiments were performed in compliance with the principles set out in the WMA Declaration of Helsinki and the Department of Health and Human Belmont Report.

### Human iPSC generation and cardiomyocyte differentiation

For iPSC generation, control and patient primary skin fibroblasts (PSF, passage 3) were infected with Sendai viruses encoding *OCT4, SOX2, KLF4,* and *c-MYC* (Life Technologies, 3 MOI each) and cultured as previously described (Moretti *et al*, 2010; Jung *et al*, 2012; Bellin *et al*, 2013).

The presence of the *TTN*-c.43628insAT mutation was verified in control and patient PSFs and iPSCs by PCR and direct sequencing using 50 ng of genomic DNA (Genomic DNA Purification Kit; Gentra Systems). Primer sequences are listed in Supplementary Table S3.

Karyotyping of the iPSC lines was performed at the Institute of Human Genetics of the Technische Universität München using standard methodology.

Assessment of pluripotency and differentiation of the iPSCs were performed as previously described (Moretti *et al*, 2010; Jung *et al*, 2012; Bellin *et al*, 2013) (see Supplementary Information for details). For PluriTest, RNA was collected from undifferentiated cells, processed using the Illumina TotalPrep RNA Amplification Kit (Life Technologies) and hybridized to the Human HT-12 v4 Expression BeadChip Kit (Illumina). Raw microarray data were uploaded to the PluriTest website (http://www.pluritest.org) and analyzed online with the published PluriTest algorithm (Muller *et al*, 2011; Bellin *et al*, 2013). For the quantification of the DNA methylation levels of *RAB25*, *NANOG*, *PTPN6*, *MGMT*, *GBP3*, and *LYST*, genomic DNA was collected from control and patient PSFs and iPSCs and the DNA methylation was assessed by quantitative real-time PCR (qRT–PCR) using the *OneStep* qMethyl Human Pluripotent Stem Cell Panel (Zymo Research) according to the manufacturer's guidelines.

Spontaneously contracting areas were manually dissected at day 20–30 of differentiation. For single-cell analysis, myocytic explants were collagenase-dissociated and plated on fibronectin-coated plates for molecular and immunocytochemical analyses.

### 2OMePS-AON transfection of HL-1 and hiPSC-derived cardiomyocytes

Transient transfection of 2OMePS-AONs (200 nM final concentration) was performed in single HL-1 and hiPSC-derived cardiomyocytes using the PEI (Fermentas) and the *Trans*IT-LT1 (Mirus) Transfection Reagent, respectively, according to the manufacturer's guidelines.

Successful excision of *titin* exon 326 was confirmed by RT–PCR and direct sequencing as described in details in the Supplementary Information.

### AON lentiviral infection of hiPSC-derived cardiomyocytes

Lentiviruses were produced in HEK293T cells by transient cotransfection of the CMVΔR8.74 packaging plasmid, the VGV.G envelope plasmid, and one of the U7snRNA-AONs-IRES-GFP lentiviral transfer vector plasmids (for cloning details see Supplementary Information) using FuGENE HD (Promega). Viral supernatants were harvested after 48 h, filtered through a 0.45-μm low-protein-binding cellulose acetate filter, and used directly to infect single iPSC-derived cardiomyocytes in the presence of 8 μg/ml polybrene. Infected cells were harvested 1 day later for the detection of *TTN* exon 326 skipping by RT–PCR and sequencing analyses and 5 days later for immunofluorescence and qRT–PCR studies.

### Mass spectrometric analysis

iPSC-derived cardiomyocytes and mouse heart tissue were lysed and experiments were performed as previously described (Michalski *et al*, 2012; Kulak *et al*, 2014). Raw mass spectrometric data were processed with MaxQuant (version. 1.4.3.19, http://www.maxquant.org) (Cox & Mann, 2008) and imported into the MaxQB database (Schaab *et al*, 2012). Bioinformatic analyses were performed with the Perseus software (http://www.perseus-framework.org). For details, see Supplementary Information.

### *Ttn* knock-in embryo studies

The knock-in (*Ttn* c.43628insAT) mouse model used was already reported (Gramlich *et al*, 2009). *Ttn* heterozygous mice were paired and checked for a vaginal plug the next morning. At day 8.5, post-coitum embryos were harvested, transferred to an agarose-coated 24-well plate with supplemented DMEM (0.5% heat inactivated FBS), and cultured under standard conditions (Hang & Chang, 2012). Genotypes were obtained from the yolk sac as described previously (Gramlich *et al*, 2009). Homozygous *Ttn* embryos were transfected with 600 nM 2OMePS-AON1 + AON3 (*n *=* *3 per group, 600 nM final concentration each) or infected with the U7snRNA-mScrAONs-IRES-GFP and the U7snRNA-m*Ttn*AONs-IRES-GFP vectors (*n *=* *7). Wild-type embryos were used as controls (*n *=* *3). Embryonic heart function was recorded with a camera, and heart function was analyzed by planimetry (Hang & Chang, 2012). Twenty-four hours later, heart function was assessed again and the embryos were processed for immunofluorescence and electron microscopy analyses as described in the Supplementary Information.

**The paper explained**

**Problem**

Frameshift mutations in the *TTN* gene encoding titin are a major cause for inherited forms of dilated cardiomyopathy (DCM), a heart disease characterized by ventricular dilatation, systolic dysfunction, and progressive heart failure. To date, there are no specific treatment options for DCM patients but heart transplantation. Exon skipping mediated by antisense oligonucleotides (AONs) and aimed at reframing transcripts is emerging as a promising therapeutic strategy. This study investigates the potential of AON-mediated exon skipping as a therapeutic strategy to restore *titin* reading frame and preserve myocardial function in DCM.

**Results**

Cardiomyocytes derived from patient-specific induced pluripotent stem cells carrying an autosomal-dominant frameshift *TTN* Ser14450fsX4 mutation in exon 326 and the DCM knock-in mice with the same mutation were treated with AONs that mask exonic splicing enhancer motifs in exon 326. Correction of *TTN* reading frame in patient-specific cardiomyocytes rescued defective myofibril assembly and stability and normalized sarcomeric protein expression. AON-treatment in *Ttn* knock-in mice improved sarcomere formation and contractile performance in homozygous embryos and prevented the development of the DCM phenotype in heterozygous animals.

**Impact**

The present study provides the first proof-of-principle evidence that disruption of the *titin* reading frame due to a truncating DCM mutation can be restored by exon skipping in both patient cardiomyocytes *in vitro* and mouse heart *in vivo*, indicating RNA-based strategies as a potential treatment option for DCM.

### Adult *Ttn* knock-in mouse experiments

For the administration of angiotensin II (Sigma), osmotic minipumps (Alzet) were placed into subcutaneous (S.C.) tissue via a midscapular incision in 3- to 4-month-old mice anesthetized with 2% isoflurane. Prior to implantation, cardiac function was assessed echocardiographically (for details, see Supplementary Information). The animals were treated with angiotensin II (1.4 mg/kg) for 14 days. The AON-treated group received vPMO-m*Ttn*AON or scrambled vPMO-mScrAON (6 mg/kg) at day 0 and 7 by intraperitoneal (I.P.) injection (vPMO-m*Ttn*AON, *n *=* *14; vPMO-mScrAON, *n *=* *10; heterozygous, angiotensin only, *n *=* *6; wild-type, angiotensin only, *n *=* *6). Doses and timing of vPMO injections were adapted from related publications (Wu *et al*, 2009). After 1 and 2 weeks, mice underwent echocardiographic examination. At the end of the experiment, heart muscle tissue was processed for morphological and molecular analyses as described in theSupplementary Information.

Animals' care was in accordance with institutional guidelines. All animal investigations were approved by the Institutional Animal Care and Use Committee, as well as by the Animal Review Board of the Eberhard Karls University, Tübingen (Regierungspraesidium Tuebingen M6/10).

### Statistical analysis

Continuous variables in different groups were compared either with Student's *t*-test if they passed tests for normality and equal variance, or by nonparametric Mann–Whitney *U*-test.

Categorial data were analyzed using chi-squared test or Fisher's exact test. Two-sided *P*-values of less than 0.05 were considered statistically significant. When not otherwise specified, error bars represent standard deviations.

## Supporting Information

Supplementary Information

Review Process File
