# Supplementary material for: Antisense-mediated exon skipping: a therapeutic strategy for titin-based dilated cardiomyopathy
Source: EMBO Mol Med. 2015 Mar 10;7(5):562–76. doi: 10.15252/emmm.201505047 (PMC4492817; doi:10.15252/emmm.201505047)
Supplement: Supplementary file 1 [file emmm0007-0562-sd1.pdf]

SUPPLEMENTARY INFORMATION

List of content:

Supplementary Materials and Methods

- Reverse transcription PCR (RT-PCR), quantitative real-time PCR (qRT-PCR) and direct sequencing
- Generation and cardiac differentiation of induced pluripotent stem cells
- Immunocytological analysis and alkaline phosphatase activity detection in iPSCs or iPSC-derived cardiomyocytes
- Generation of the U7snRNA lentiviral vectors
- Mass Spectrometry sample preparation and data processing
- Live-cell imaging in iPSC-derived cardiomyocytes
- Immunohystological and electron microscopy analyses in *Ttn* knock-in embryos
- Echocardiographic analysis in adult *Ttn* knock-in mice
- Heart morphological and molecular analyses in adult *Ttn* knock-in mice

Supplementary Results

- Characterization of control and DCM induced pluripotent stem cells
- Sarcomere remodelling in actin-RFP transfected iPSC-derived cardiomyocytes

Supplementary Tables

Supplementary Table S1. AON sequences.

Supplementary Table S2. List of primers for nested PCR.

Supplementary Table S3. Primers for RT-PCR, mutagenesis PCR, and sequencing.

Supplementary Figures

Supplementary Figure S1. Assessment of AON expression and stability after transfection in HL-1 cardiomyocytes.

Supplementary Figure S2. Location of primers for the nested PCR.

Supplementary Figure S3. Generation and characterization of DCM iPSCs.

Supplementary Figure S4. Assessment of pluripotency in control and DCM iPSC clones.

Supplementary Figure S5. Transfection efficiency of iPSC-derived cardiomyocytes.

Supplementary Figure S6. Efficient skipping of *TTN* exon 326 in control iPSC-derived cardiomyocytes.

Supplementary Figure S7. Live-cell imaging of control iPSC-derived cardiomyocytes overexpressing an actin-RFP fusion protein.

Supplementary Figure S8. Effect of lentiviral infection on sarcomere remodeling in control and DCM iPSC derived cardiomyocytes.

Supplementary Figure S9. *In vivo* evaluation of 2OMePS and vivo-morpholino (vPMO) modified antisense oligonucleotides.

Supplementary References

Supplementary Materials and Methods

Cell lines

The HL-1 cells were obtained from Dr. W. Claycomb, Departments of Biochemistry and Molecular Biology, Louisiana State University Medical Center, New Orleans.

Control primary skin fibroblasts (PSFs) were obtained from a healthy donor's skin biopsy at the Klinikum rechts der Isar, Technische Universität München. DCM PSFs were obtained from the patient's skin biopsy at the Royal Brisbane and Women's Hospital, Brisbane, Australia. Before performing experiments, all the cell lines were tested for mycoplasma contamination.

Reverse transcription PCR (RT-PCR), quantitative real-time PCR (qRT-PCR) and direct sequencing

Total mRNA was isolated from PSF, iPSCs, EBs, and cardiomyocytes using the Stratagene Absolutely RNA kit and 1µg was used to synthesize cDNA using the High-Capacity cDNA Reverse Transcription kit (Applied Biosystems). Gene expression was quantified by qRT-PCR using 1µl of the RT reaction and the Power SYBR Green PCR Master Mix (Applied Biosystems). Gene expression levels were normalized to *GAPDH*.

To confirm successful excision of *Titin* exon 326**,** 1µg of RNA was used for cDNA synthesis (Transcriptor first strand cDNA synthesis kit, Roche Applied Science) with a *titin* specific reverse primer in exon 328 (ex238r). A nested PCR was established with two external (ex324f and 328r) and 2 internal primers (ex325f and ex327r for amplification of the skipped allele, and ex325f and ex326r for the unskipped allele) using the Expand Long Template PCR System (Roche Applied Science). Sanger sequencing of the exon transition zones was performed with primer ex325f at Seqlab (Göttingen, Germany) for the *Ttn* mouse gene and at Eurofins MWG Operon (Munich, Germany) for the human *TTN* gene. Primer localization is shown in Fig S2 and mouse and human primer sequences are listed in Table S2 and Table S3.

Generation and cardiac differentiation of induced pluripotent stem cells

Control and patient PSFs were infected with Sendai viruses encoding *OCT4, SOX2, KLF4,* and *c-MYC* (Life Technologies, 3 MOI each), seeded on irradiated on murine embryonic feeder cells until iPSC colonies could be picked and, after 4 days, trypsinized and seeded on irradiated mouse embryonic fibroblasts (MEFs) in human embryonic stem cell (hESC) medium consisting of DMEM/F12 supplemented with 20% knockout serum replacement (KSR, Invitrogen), 2mM L-glutamine, 0.1mM nonessential amino acids, 0.1mM β-mercaptoethanol, 50U/ml penicillin, 50mg/ml streptomycin and 10ng/ml human b-FGF

(R&D). After derivation, iPSCs were grown on matrigel (BD) in MEF-conditioned hESC medium and enzymatically passaged using 1 mg/ml dispase (Stem Cell Technologies).

Control and patient iPSCs were differentiated as embryoid bodies (EBs) by dissociation of iPSC colonies with dispase and maintaining them for 3 days in MEF-conditioned hESC medium in low attachment plates coated with 5% (w/vol) poly-HEMA (Sigma-Aldrich). For spontaneous differentiation, medium was then replaced with DMEM/F12 supplemented with 20% FBS, 2mM L-glutamine, 0.1mM nonessential amino acids, 0.1mM β-mercaptoethanol, 50U/ml penicillin, and 50mg/ml streptomycin. To improve cardiac differentiation, ascorbic acid (50mg/ml) was added to the medium and EBs were plated on day 7 on gelatin-coated dishes for better detection of beating foci. At day 20-30 of EB differentiation, contracting areas were manually micro-dissected and plated on fibronectin-coated plates. These myocytic explants were maintained in culture in the same medium described for EB differentiation, but supplemented with 2% FBS.

For single-cell analysis, myocytic explants were dissociated with several rounds of 15 min incubation at 37 °C with 480U/ml collagenase type II (Worthington) under gentle shaking. Dissociated cells were plated on fibronectin-coated plates and maintained in EB differentiation medium supplemented with 2% FBS. For Isoprotenerol treatment, 10μM Isoprotenerol (ISO, Sigma-Aldrich) was added to medium. Medium was changed every second day and after one week cells were processed for molecular and immunocytochemical analyses.

Before performing experiments, all the cell lines were tested for mycoplasma contamination.

Immunocytological analysis and alkaline phosphatase activity detection in iPSCs or iPSC-derived cardiomyocytes

iPSCs or iPSC-derived cardiomyocytes were fixed in 3.7% (vol/vol) formaldehyde and subjected to immunostaining by using the following primary antibodies: human Nanog (rabbit polyclonal, Abcam, ab106465, 1:500), TRA1-81-Alexa-Fluor-488-conjugated (mouse monoclonal, 560174, BD Pharmingen, 1:20), cardiac troponin T (mouse monoclonal clone 13-11, Lab Vision, 1:500), α-actinin (mouse monoclonal clone EA-53, Sigma-Aldrich, 1:300), Z-disc Titin (rabbit polyclonal, 1:50, kindly provided by Prof. Labeit), SRF (rabbit polyclonal G-20, Santa Cruz, 1:50), MURF2 (rabbit polyclonal, 1:100, kindly provided by Prof. Labeit), Nbr1 (rabbit monoclonal, clone D2E6, Cell Signaling, 1:400) and SQSTM1/p62 (rabbit monoclonal, clone D10E10, Cell signalling, 1:400). Alexa-Fluor-488, -594, and -647 conjugated secondary antibodies specific to the appropriate species were used (Life

Technologies, 1:500). Nuclei were detected with 1 μg/ml Hoechst 33528. Direct alkaline phosphatase activity was analysed using the NBT/BCIP alkaline phosphatase blue substrate (Roche), according to the manufacturer's guidelines.

Microscopy was performed using imaging systems (DMI6000-AF6000), filter cubes and software from Leica microsystems. Images were assigned with pseudo-colors. Morphological analyses were performed by investigators blinded to the genotype of the cells.

Generation of the U7snRNA lentiviral vectors

For the generation of the lentiviral vectors we used as template a previously described lentiviral vector that encodes a modified U7 small nuclear RNA targeting exon 51 of the dystrophin gene (Goyenvalle et al, 2009). We exchanged the antisense sequences targeting exon 51 of dystrophin, with antisense sequences targeting two Exonic Splicing Enhancers in exon 326 of human *TTN* (U7snRNA-*TTN*AONs-IRES-GFP vector) or mouse *Ttn* (U7snRNA-m*Ttn*AONs-IRES-GFP vector) by PCR mutagenesis, as previously described(Goyenvalle, 2012). Scrambled sequences corresponding to the human and mouse *titin* AONs were also exchanged by PCR mutagenesis (for the U7snRNA-ScrAONs-IRES-GFP and U7snRNA-mScrAONs-IRES-GFP vectors). Then the four modified U7snRNA constructs were separately cloned into the lentiviral transfer vector plasmid pRRLsin18.PPT.PGK.IRES.GFP (IRES-GFP vector). The primers used for PCR mutagenesis are listed in Table S3.

Mass Spectrometry sample preparation and data processing

Mouse heart tissue was homogenized in liquid nitrogen. Both iPSC-derived cardiomyocytes and mouse heart tissue were resuspended in a lysis buffer containing 6M guanidinium chloride (GCl) and subsequently reduced and alkylated prior to overnight digestion with LysC and Trypsin, as previously described (Kulak et al, 2014). Experiments were performed in technical triplicates using a nanoflow HPLC system (Proxeon/Thermo Fisher Scientific) coupled via a nanoelectrospray ion source (Thermo Fischer Scientific) to a Q Exactive mass spectrometer (Thermo Fisher Scientific), as previously described (Michalski et al, 2012; Kulak et al, 2014). Raw MS files were processed with MaxQuant (version. 1.4.3.19, http://www.maxquant.org) (Cox & Mann, 2008). Peak list files were searched by the Andromeda search engine, incorporated into the MaxQuant framework (Cox et al, 2011) against the UniProt database containing forward and reverse sequences. To match identifications across different replicates the 'match between runs' option in MaxQuant was enabled in a time window of two minutes. Processed data were imported into the MaxQB database (Schaab et al, 2012) and identified titin peptides were aligned to the sequence of

isoform Q8WZ42 (34350 aminoacids) for human and A2ASS6 (35213 aminoacids) for mouse samples. 298 peptides were annotated as mapping in the exon 326 in human samples (corresponding to aminoacids 21598-27300) and 401 in mouse samples (corresponding to aminoacids 22449-28161). All peptides downstream of the exon 326 were annotated as C-terminal. Bioinformatic analyses were performed with the Perseus software (www.perseus-framework.org). Before hierarchical clustering, peptide intensities were logarithmized and normalized by subtracting the median value. Data were filtered for at least 50% valid values.

To determine the *in vivo* skipping efficiency, the intensities of C-terminal peptides were summed and relative intensity was calculated as percent of total intensity of all mouse titin peptides (from 1 to 35213). The number of peptides used for this analysis was 2129 for total titin and 490 for the C-terminal portion. In vPMO-mScrAON-treated mice, the relative amount of the C-terminal portion resulted as 18.7% of total titin intensity while in vPMO-m*Ttn*AON animals resulted as 20.15% (n=3, P=6.23E-3, two-sided Student´s T-test). Using this C-terminal increase as a proxy to quantitate exon skipping at the protein level, we thus estimated, based on the simple proportion [18.70:100=20.15:x], that the *in vivo* skipping efficiency was around 8%.

Live-cell imaging in iPSC-derived cardiomyocytes

Control myocytic explants were dissociated, plated on fibronectin-coated plates and infected with the CellLight® Actin-RFP baculovirus (Life Technologies, 10 MOI). The medium was changed after 24 hours and then every second day. Cells were monitored every day for two weeks. Microscopy was performed using imaging systems (DMI6000-AF6000), filter cubes and software from Leica microsystems. Images were assigned with a pseudo-color.

Immunohystological and electron microscopy analyses in Ttn knock-in embryos

Mouse embryos were fixed in 3.7% (vol/vol) formaldehyde and subjected to immunostaining by using the following primary antibodies: α-actinin (Sigma-Aldrich) and myosin heavy chain (Chemicon). Alexa-Fluor-488 and -594 conjugated secondary antibodies specific to the appropriate species were used (Life Technologies, 1:500). Nuclei were detected with DAPI.

For electron microscopy, mouse embryos were fixed with 3% formaldehyde in 0.2M Hepes, pH 7.4, for 30 min, followed by immersion in 6% formaldehyde/0.1% glutaraldehyde in 0.2M Hepes, pH 7.4, overnight. They were postfixed with 1% OsO_4 for 2h, dehydrated in a graded ethanol series and propylene oxide and embedded in Poly/Bed^R 812 (Polysciences, Inc., Eppelheim, Germany).

Semithin sections of the heart region were stained with toluidine blue. Ultrathin sections (70 nm) were contrasted with uranyl acetate and lead citrate and examined with a Zeiss 910 electron microscope. Digital images were taken with a 1kx1k high-speed slow scan CCD camera (Proscan).

Echocardiographic analysis and vPMO treatment in adult Ttn knock-in mice

After anesthesia induction with 2% isoflurane in an anesthesia chamber, 3-4 months old male mice (B6.129P2-*Ttn*^{tm1Mdc}; strain: C57BL/6; genetic modification: *Ttn* c.43628insAT) were placed on a heated, tilt platform for echocardiography. Body temperature was maintained at 37°C, and anesthesia was continued with 0.5-1.5% isoflurane. An echocardiograph system (Vevo 2100, VisualSonics, Toronto, ON, Canada) with a 30-MHz scanhead was used. Images were collected and stored as a digital cine loop for off-line calculations. Standard imaging planes, M-mode, Doppler, and functional calculations were obtained according to American Society of Echocardiography guidelines. The parasternal long-axis four-chamber view of the left ventricle was used to guide calculations of percentage fractional shortening, percentage ejection fraction, and ventricular dimensions and volumes. The subcostal long-axis view from the left apex was used to obtain mitral inflow and aortic ejection profiles by pulsed-wave Doppler imaging. Echocardiographic analysis was performed by investigators blinded to the genotype of the animals.

In the adult mice study, the central hypothesis was that in the Angiotensin-treated animals, vPMO-m*Ttn*-AON admnistration results in an improved LV-EF compared to vPMO-mScr injection. Assuming a standard deviation of 12% of the echocardiographically-determined LV-EF, in order to demonstrate 15% LV-EF increase with a type I error rate of 5% and with 90% power, we estimated that we would need a sample size of ~11 animals per group. The mice were matched by age, body weight and genotype and then randomly allocated to the different treatment groups. 14 mice received the vPMO-m*Ttn*-AON and 11 the vPMO-mScr; one vPMO-m*Ttn*-AON-treated animal died and was excluded from the study.

Housing and husbandry conditions were in accordance to the PHS Policy on Humane Care and Use of Laboratory Animals and were monitored and enforced by the Institutional Animal Care and Use Committee of Eberhard Karls University, Tübingen. According to the ARRIVE guidelines all relevant aspects of the study were reported. All animal investigations were done in compliance with the NIH and MRC recommendations.

Heart morphological and molecular analyses in adult Ttn knock-in mice

At the end of two weeks angiotensin II treatment mice were sacrified and heart muscle tissue was fixed in 4% paraformaldehyde overnight, dehydrated through a gradient of alcohol and embedded in paraffin. Sections (8 µm) were prepared and stained with Masson's trichrome. For expression analysis, RNA was extracted, reverse transcribed and analyzed as described. Successful penetration of vPMOs into the cardiomyocytes was demonstrated by fluorescence in situ hybridization (FISH). Slides were fixed in 4% PFA for 10 minutes and permeabilized in 0.2mg/ml Saponin (47036, Sigma-Alidrich) for 20 minutes at room temperature. Slides were postfixed in 4% PFA for 10 minutes at room temperature and treated in acetic anhydride/triethanolamine, after rinsed with PBS, followed by 3% H_2O_2/PBS for 1 hour at room temperature. Hybridization buffer (50% Formamide, 5x SSC, 0.5mg/ml yeast tRNA, 0.5mg/ml Herring sperm DNA, 1x Denhardt's solution) was heated at 80°C for 10 minutes and the slides were pre-hybridized at 45°C for 1 hour. 100 nM locked nucleic acid (LNA) modified oligonucleotide probe complementary to the antisense Vivo-Morpholino (sequence: AGGACCACCTTCAAATGCACATGTCACT) and LNA scrambled control probe (sequence: TGTTGGGAGGCATATACATTGCCTGGA) double labelled with DIG were hybridized to the slides at 45°C over night. The hybridization solution was removed by thoroughly rinsing in 4× SSC, followed by SSC washes. The ISH signal was amplified with tyramide signal amplification kit (TSA Plus Biotin Kit, Perkin Elmer) according to the manufacture's manual and the signal was detected with Streptavidin-Fluorescein (NEL720001EA, Perkin Elmer). Animals′ care was in accordance with institutional guidelines. Mice were randomly allocated to the different treatment groups and no animals were excluded from the analysis. Analyses were performed by investigators blinded to the genotype of the mice.

Supplementary Results

Characterization of control and DCM induced pluripotent stem cells

All the induced pluripotent stem cell (iPSC) lines showed human embryonic stem cell morphology, alkaline phosphatase activity (Fig S3B), loss of Sendai viral transgenes (Fig S3C), immunoreactivity for the embryonic stem-cell markers NANOG and TRA1-81 (Fig S3D) and normal karyotype (Fig S3E); moreover they passed the so called PluriTest with a high "pluripotency score" and a low "novelty score" resembling normal human pluripotent stem cells (Fig S3F). All the iPSC lines showed reactivation of endogenous pluripotency genes (OCT4, SOX2, NANOG, REX1 and TDGF1, Fig S4A) and exhibited low DNA methylation levels of *RAB25*, *NANOG* and *PTPN6* (Nishino et al. 2011; Chamberlain et al 2010) compared to their corresponding PSFs; conversely *MGMT*, *GBP3* and *LYST* of methylation levels were higher in iPSCs than in PSFs, as expected (Nishino et al. 2011; Calvanese et al. 2008, Fig S4B). Pluripotency of each cell line was further confirmed by up-regulation of genes specific of the three germ layers during *in vitro* spontaneous differentiation into embryoid bodies (EBs, Figure S4C).

Sarcomere remodelling in actin-RFP transfected iPSC-derived cardiomyocytes

During enzymatic dissociation into single cells, iPSC-derived cardiomyocytes, differently from adult cardiomyocytes, undergo a disarray of the myofibrils and round up. As soon as the cells are plated and attach to the culture dish, they start to reorganize the myofibrils, similarly to neonatal cardiomyocytes (Atherton et al, 1986). However, with the time in culture as single cells, a certain disorganization of the newly built sarcomeres can occur in some cardiomyocytes that undergo a de-differentiation process. In order to monitor myofibril remodeling in single iPSC-derived cardiomyocyte after dissociation, we overexpressed actin as a fusion with a red fluorescent protein (RFP) in freshly dissociated cells by Baculovirus technology (Fig S7A) and followed over time the distribution pattern of the fluorescent actin molecules in single cardiomyocytes. This live cell imaging analysis of many single control iPSC-derived cardiomyocytes up to 2 weeks after dissociation revealed that, similarly to neonatal cardiomyocytes, there is a considerable heterogeneity in the degree of myofibril organization at day 1-2, but most of the cells show organized RFP+ sarcomeres mainly around the nucleus (Fig S7B), suggesting this as starting site of myofibril reassembly. Within 4-5 days, the myofibril organization extends to the whole cell (Fig S7B) and this associates with the onset of rhythmic contraction, supporting previously reported data on sarcomerogenesis in rodent embryonic cardiomyocytes (Turnacioglu et al, 1997; Sparrow &

Schock, 2009). We considered cells at this stage as “fully organized”. By following them further we noticed that, around 7-10 days of culture as single cells, few of the “fully organized” cardiomycytes began to lose their sarcomeric organization, starting at the perinuclear region and eventually throughout the whole cytoplasm till the cell periphery (Fig S7B), resembling the pattern observed during sarcomere disassembly in dividing mammalian cardiomyocytes (Ahuja et al, 2004). We saw this phenomenon in hundreds of cells through many independent experiments. Based on these observations, we hypothesized that sarcomere remodeling in iPSC-derived cardiomyocytes after dissociation is a radially control process that mimics the one earlier observed in neonatal cardiomyocyte (Atherton et al, 1986). Thus, we assumed that cells with organized myofibrils in the perinuclear region are somehow in the process of sarcomere assembly, while cells with a striated pattern only in the cell periphery are in the process of sarcomere disassembly.

Supplementary Tables

Supplementary Table S1: AON sequences

Name	Sequence
2OMePS- AON1	UGC AUUUGA AGG UGG UCC U
2OMePS- AON2	CCG AUU UCU UGG UGG UAU CA
2OMePS- AON3	AUC CAG GCA UCA UCU CCU AC
2OMePS- AON4	UUG AUC CAG GCA UCA UCU CCU
2OMePS- hAON1	UUG AUG CUC CAC GAC AUA G
2OMePS- hAON3	CCA UGC CAA AGA AGC AGA UU
vPMO-AON1	AGU GAC AUG UGC AUU UGA AGG UGG UCC U

Supplementary Table S2: List of primers for nested PCR

Name	Species	Sequence
ex324f	Mouse	ATACCTCTTCCGTGTGTCAGCAG
ex325f	Mouse	ACCAGTTTCCGATCTCAGGTGC
ex326r	Mouse	TTCAACATTTGGAATCACAGC
ex327r	Mouse	TTGATGAGGACAGGAAGCAG
ex328r	Mouse	ACTTTAATCATGGTTCTTGCTACTG
hex324f	Human	GTACATCTTCCGGGTCTCAGCTG
hex325f	Human	ACCAGTTTCCGATCTCAGGTGC
hex326r	Human	TTCAACATCTGGAATCACCGC
hex327r	Human	TTAATAAGAACAGGAAGCAG
hex328r	Human	ACTTTCATCATGGTTCTTGCAACTG

Supplementary Table S3: Primers for RT-PCR, mutagenesis PCR, and sequencing

Name	Use	Sequence	
hTTNe326AT	Sequencing	For	CCTCTGAAGCACCATCTCCAC
		Rev	CCTTCCAAGTGATGGCAGTG
Sendai Virus	qRT-PCR	For	GGATCACTAGGTGATATCGAGC
		Rev	ACCAGACAAGAGTTTAAGAGATATGTA TC
hGAPDH	qRT-PCR	For	TCCTCTGACTTCAACAGCGA
		Rev	GGGTCTTACTCCTTGGAGGC
hc-MYC endo	qRT-PCR	For	AGAAATGTCCTGAGCAATCACC
		Rev	AAGGTTGTGAGGTTGCATTTGA
hKLF4 endo	qRT-PCR	For	ATAGCCTAAATGATGGTGCTTGG
		Rev	AACTTTGGCTTCCTTGTTTGG

hOCT3/4 endo	qRT-PCR	For	GACAGGGGGAGGGGAGGAGCTAGG
		Rev	CTTCCCTCCAACCAGTTGCCCCAAAC
hSOX2 endo	qRT-PCR	For	GGGAAATGGGAGGGGTGCAAAAGAGG
		Rev	TTGCGTGAGTGTGGATGGGATTGGTG
hNANOG	qRT-PCR	For	TGCAAGAACTCTCCAACATCCT
		Rev	ATTGCTATTCTTCGGCCAGTT
hREX1	qRT-PCR	For	ACCAGCACACTAGGCAAACC
		Rev	TTCTGTTCACACAGGCTCCA
hTDGF1	qRT-PCR	For	CCCAAGAAGTGTTCCCTGTG
		Rev	ACGTGCAGACGGTGGTAGTT
hPDX1	qRT-PCR	For	AAGCTCACGCGTGGAAAG
		Rev	GGCCGTGAGATGTACTTGTTG
hSOX7	qRT-PCR	For	TGAACGCCTTCATGGTTTG
		Rev	AGCGCCTTCCACGACTTT
hAFP	qRT-PCR	For	GTGCCAAGCTCAGGGTGTAG
		Rev	CAGCCTCAAGTTGTTCCTCTG
hCD31	qRT-PCR	For	ATGCCGTGGAAAGCAGATAC
		Rev	CTGTTCTTCTCGGAACATGGA
hDES	qRT-PCR	For	GTGAAGATGGCCCTGGATGT
		Rev	TGGTTTCTCGGAAGTTGAGG
hACTA2	qRT-PCR	For	GTGATCACCATCGGAAATGAA
		Rev	TCATGATGCTGTTGTAGGTGGT
hSCL	qRT-PCR	For	CCAACAATCGAGTGAAGAGGA
		Rev	CCGGCTGTTGGTGAAGATAC
hMYL2	qRT-PCR	For	TACGTTCGGGAAATGCTGAC
		Rev	TTCTCCGTGGGTGATGATG
hCDH5	qRT-PCR	For	GAGCATCCAGGCAGTGGTAG
		Rev	CAGGAAGATGAGCAGGGTGA
hKRT14	qRT-PCR	For	CACCTCTCCTCCTCCCAGTT
		Rev	ATGACCTTGGTGCGGATTT
hNCAM1	qRT-PCR	For	CAGATGGGAGAGGATGGAAA
		Rev	CAGACGGGAGCCTGATCTCT
hTH	qRT-PCR	For	TGTACTGGTTCACGGTGGAGT
		Rev	TCTCAGGCTCCTCAGACAGG
hGABRR2	qRT-PCR	For	CTGTGCCTGCCAGAGTTTCA
		Rev	ACGGCCTTGACGTAGGAGA
hMYH6	qRT-PCR	For	TCAGCTGGAGGCCAAAGTAAAGGA
		Rev	TTCTTGAGCTCTGAGCACTCGTCT
hMYH7	qRT-PCR	For	TCGTGCCTGATGACAAACAGGAGT

		Rev	ATACTCGGTCTCGGCAGTGACTTT
hACTC1	qRT-PCR	For	CCAAGATGTGTGACGACGAG
		Rev	ACGATGGACGGGAAGACA
U7-*TTN*326	Mutagenesis PCR	For	ACATAGCCATGCCAAAGAAGCAGATTAATTTTTGG AGCAGGTTTTCT
		Rev	TTGGCATGGCTATGTCGTGGAGCATCAATTGCGGA AGTGCGTCTGTA
U7-*TTN*Scr326	Mutagenesis PCR	For	TTCTTAGATACAATCAACCGGCATGAAATTTTTGGA GCAGGTTTTCT
		Rev	GATTGTATCTAAGAAGTTCGTGCTGCTACGCTTGCG GAAGTGCGTCTGTA
U7-m*Ttn*326	Mutagenesis PCR	For	CTCCTACTGCATTTGAAGGTGGTCCTAATTTTTGGA GCAGGTTTTCT
		Rev	CAAATGCAGTAGGAGATGATGCCTGGATTTGCGGA AGTGCGTCTGTA
U7-m*Ttn*Scr326	Mutagenesis PCR	For	ACTACCAATGTGGCTCGTCGTAATTGAATTTTTGGA GCAGGTTTTCT
		Rev	AGCCACATTGGTAGTCGAGATATGGAGCTTGCGGA AGTGCGTCTGTA
U7-Titin326ext	Mutagenesis PCR	For	GGGATCGATTAACAACATAGGAGCTGTG
		Rev	AAAGATATCCACATACGCGTTTCCTAGGA

Supplementary Figures

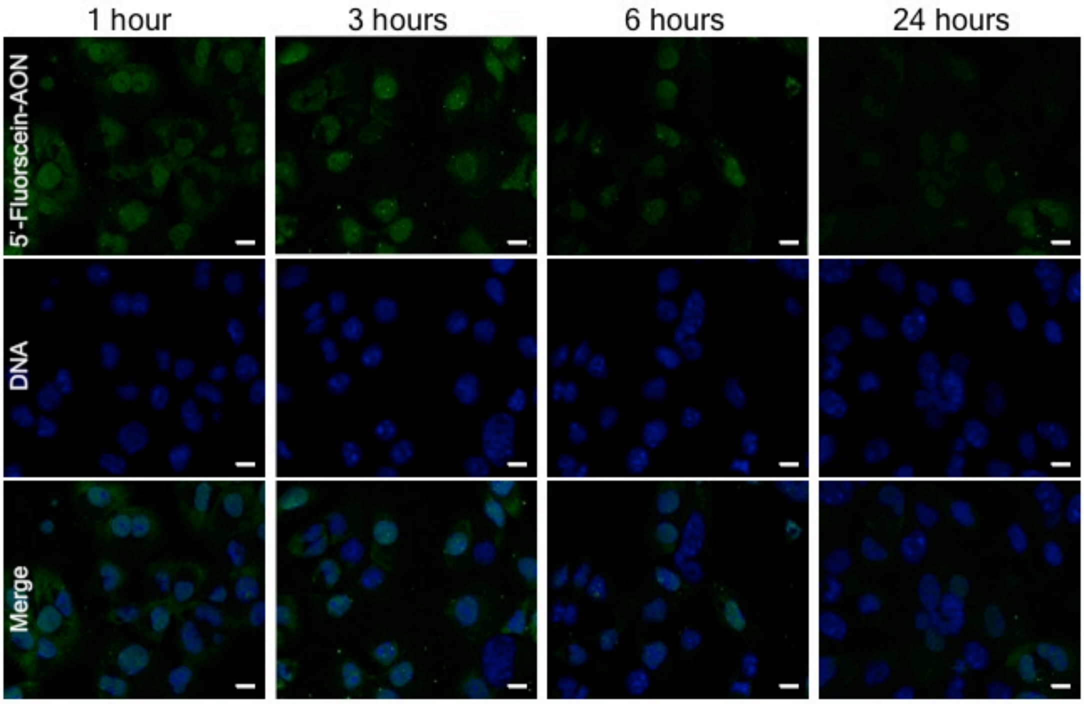

Supplementary Figure S1. Assessment of AON expression and stability after transfection in HL-1 cardiomyocytes.

Representative images of HL-1 cardiomyocytes after transfection of a 5'-fluorescein-labeled 2OMePS AON (green) at the indicated time points. Fluorescence signal in the nucleus peaks approximately after 3h transfection. Scale bar, 10μm.

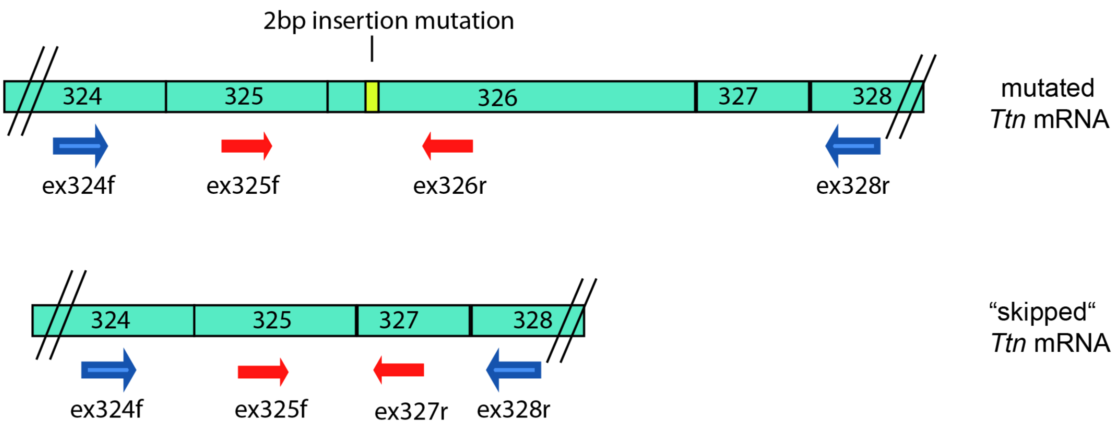

Supplementary Figure S2. Location of primers for the nested PCR.
This figure shows a schematic of the primers used for detection of successful removal of *titin* (*Ttn*) exon 326 from the transcript. An external long range PCR (using the primer pairs depicted in blue) followed by an internal nested PCR (using the red primer pairs) discriminate between unskipped (wild-type and mutated) and skipped *Ttn* mRNA. Due to the size of exon 326, exon 325-326 and exon 325-327 were assessed by separate primers pairs, ex325f-ex326r and ex325f-ex327r, respectively. Exact primer sequences are listed in Supplementary Table S2.

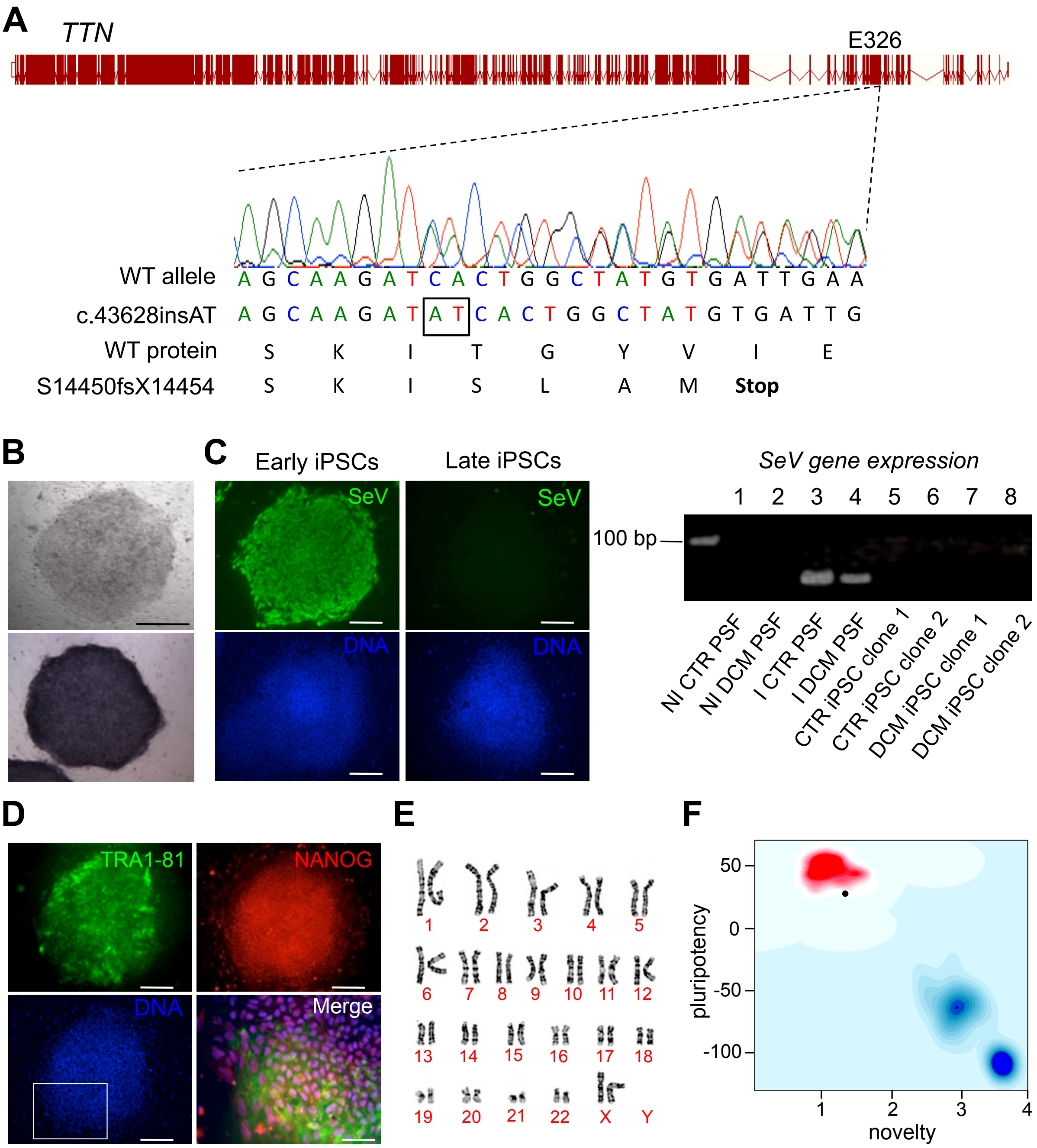

Supplementary Figure S3. Generation and characterization of DCM iPSCs.

A, Sequencing results of the human *Titin* (*TTN*) gene in a representative DCM iPSC clone, revealing an heterozygous AT insertion mutation (c.43428insAT) in exon 326. This mutation causes a frameshift, generating a premature stop codon after addition of four novel amino acid residues (S14450fsX14454). **B**, Images of colonies from a representative DCM iPSC clone in bright field (top) and after staining for alkaline phosphatase (AP) activity (bottom). Scale bar, 500µm. **C**, Sendai virus (SeV) immunostaining of two colonies from the same DCM iPSC clone at early and later culture passages (right). Scale bar, 200µm. RT-PCR for the expression of SeV transgenes in two control (CTR) and two DCM iPSC clones at passage 6 (left). NI= not infected; I=infected. **D**, Immunofluorescence analysis of pluripotency markers NANOG (red) and TRA1-81 (green) in a representative DCM iPSC clone. Scale bar, 200µm. Merged image is a magnification of the area framed in the adjacent panel. Scale bar, 50µm. **E**, Karyotyping of a representative DCM iPSC clone. **F**, PluriTest analysis of DCM iPSCs (black circle) with a high "pluripotency score" and a low "novelty score" indicating that they resemble normal human pluripotent stem cells (in red).

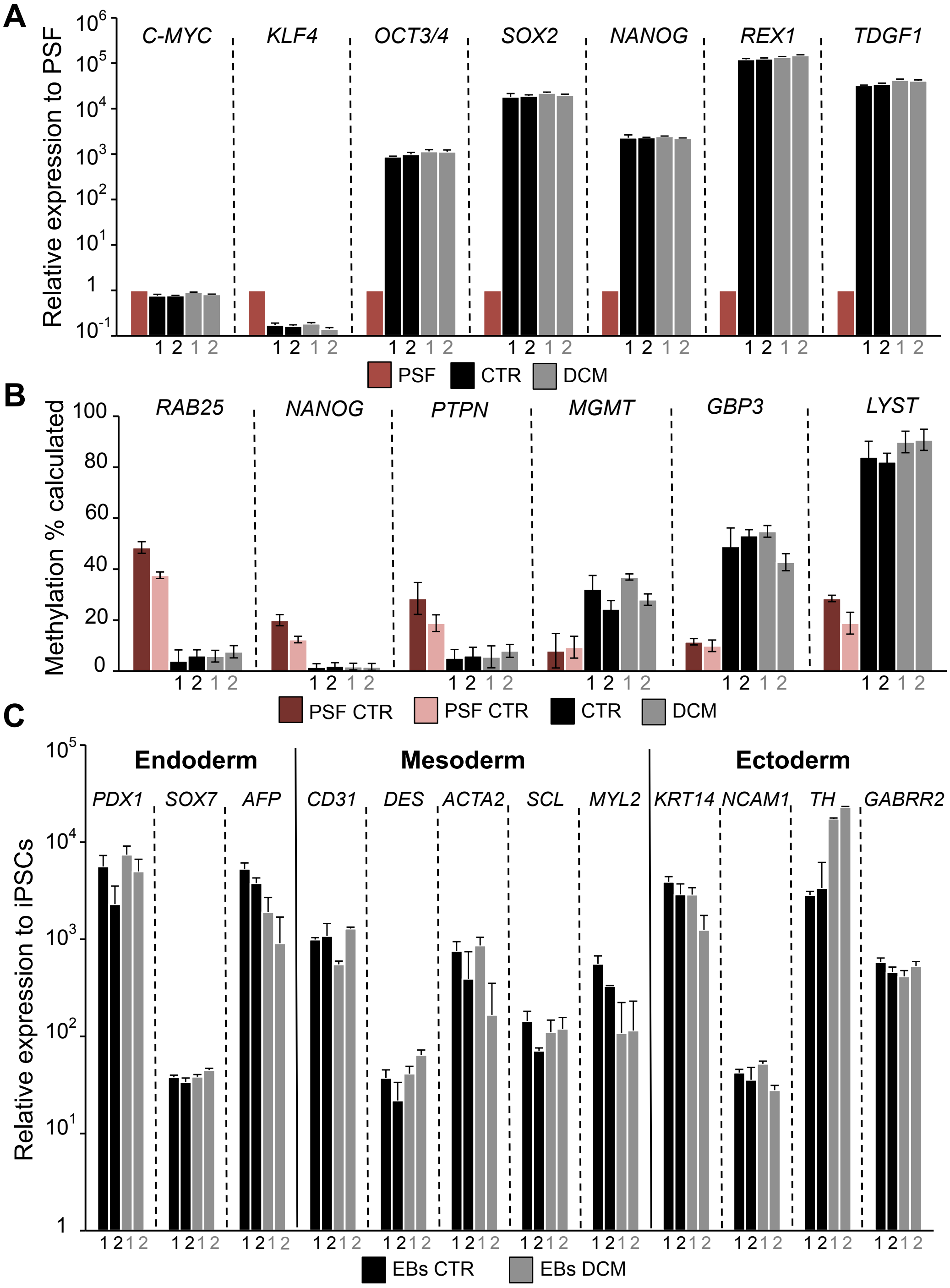

Supplementary Figure S4. Assessment of pluripotency in control and DCM iPSC clones. **A**, Quantitative RT-PCR (qRT-PCR) analysis of endogenous genes associated with pluripotency (c-MYC, KLF4, OCT3/4, SOX2, NANOG, REX1, and TDGF1) in two control (1 and 2, black bars) and two DCM iPSC clones (1 and 2, grey bars). Expression values are relative to corresponding primary skin fibroblasts (PSF, red bars), normalized to GAPDH, and presented as mean ± s.e.m., *n*=3. **B**, qRT-PCR analysis of DNA methylation levels of *RAB25*, *NANOG*, *PTPN6*, *MGMT*, *GBP3* and *LYST* in two control (1 and 2, black bars), two DCM iPSC clones (1 and 2, grey bars) and corresponding PSFs (dark red and pink bars). Values are presented as mean ± s.e.m., *n*=3. **C**, qRT-PCR analysis of markers of the three germ layers, endoderm (PDX1, SOX7, and AFP), mesoderm (CD31, DES, ACTA2, SCL, MYL2, and CDH5), and ectoderm (KRT14, NCAM1, TH, and GABRR2) in embryoid bodies (EBs) at day 21 of differentiation from two control (1 and 2, black bars) and two DCM iPSC clones (1 and 2, grey bars). Expression values are relative to corresponding undifferentiated iPSC clones, normalized to GAPDH, and presented as mean ± s.e.m., *n*=3.

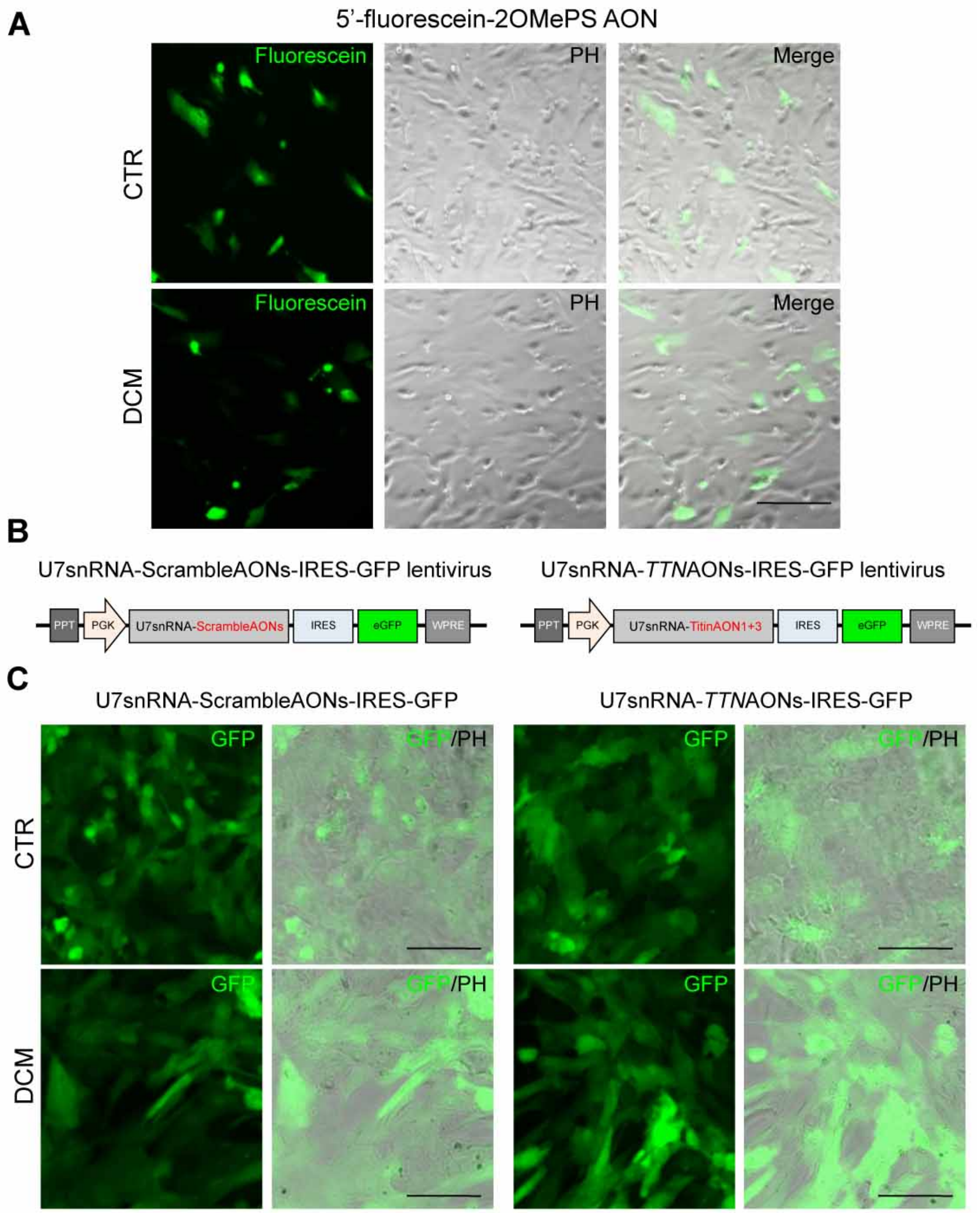

Supplementary Figure S5. AON transfection efficiency of iPSC-derived cardiomyocytes. **A**, iPSC-derived control and DCM cardiomyocytes were transfected with 5'-fluorescein-labeled 2OMePS AON (600 nM final concentration) and imaged 3 hrs after. Scale bar, 200μm. **B**, Schematic of the U7snRNA-*TTN*AONs-IRES-GFP and U7snRNA-ScrambleAONs-IRES-GFP (control) lentiviral vectors. For the expression of the AON1 and AON3, AONs was inserted into the U7snRNA-IRES-GFP vector, a scrambled control was made in the same way. **C**, GFP expression in iPSC-derived cardiomyocytes after lentiviral infection. Images were acquired 24 hrs after infection. Scale bar, 100μm.

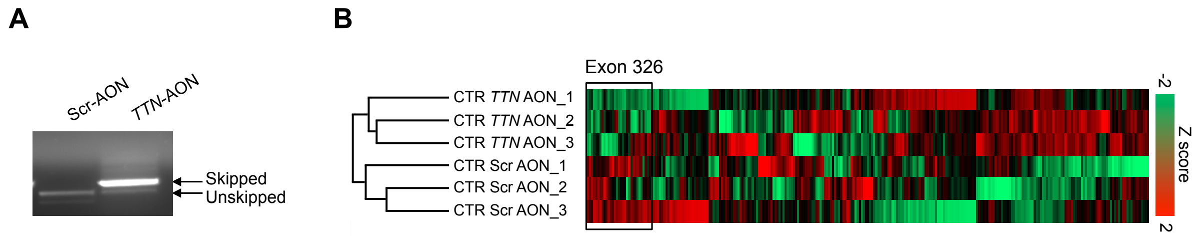

Supplementary Figure S6. Efficient skipping of *TTN* exon 326 in control iPSC-derived cardiomyocytes.

A, RT-PCR and **B**, mass spectrometric analysis of TTN peptides in control iPSC-derived cardiomyocytes infected with the U7snRNA-ScrAONs-IRES-GFP and U7snRNA-*TTN*AONs-IRES-GFP lentiviral vectors. In B, hierarchical clustering showed the presence of a cluster significantly enriched (n=3, P=1.99E-02, Fischer's exact test, FDR=0.04) in exon 326 peptides that was significantly down-regulated in CTR *TTN* AON cardiomyocytes compared to CTR Scr AON cardiomyocytes. Statistical difference was tested using the two-sided Fischer's exact test.

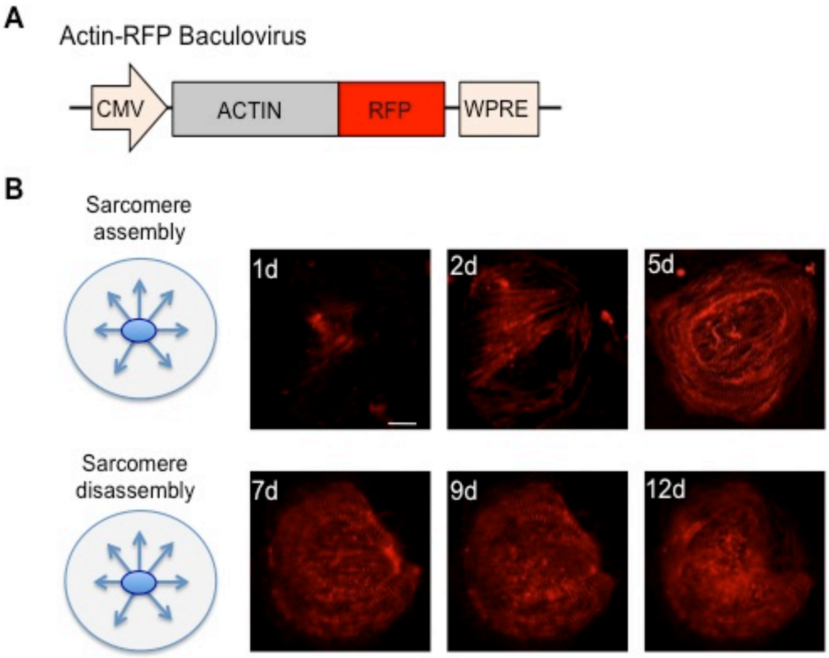

Supplementary Figure S7. Live cell imaging of control iPSC-derived cardiomyocytes overexpressing an actin-RFP fusion protein.

A, Schematic representation of the modified insect viral (baculovirus) vector containing an actin-red fluorescent fusion protein construct. **B**, Images from a representative infected single control iPSC-derived cardiomyocyte in the process of sarcomere assembly (top) and disassembly (bottom), respectively. Two days after infection the cell exhibits mature myofibrils with regular cross-striations only in the perinuclear region. By day 5, striated sarcomeres occupy the whole cytoplasm. The myofibril organization is progressively lost by time in culture, with sarcomere disassembling starting around the nucleus and proceeding towards the periphery of the cell. We observed this temporal progression in sarcomere remodeling in numerous cells, indicating that in enzymatically dissociated hiPSC-derived cardiomyocytes formation of a complete myofibril network requires roughly a week. With progressive culture some cells start to spontaneously disassemble their myofibrils. Both processes of sarcomere assembly and disassembly in mature myofibrils are radially occurring and progress temporally from the center to the edges of the cell. Scale bar, 25μm.

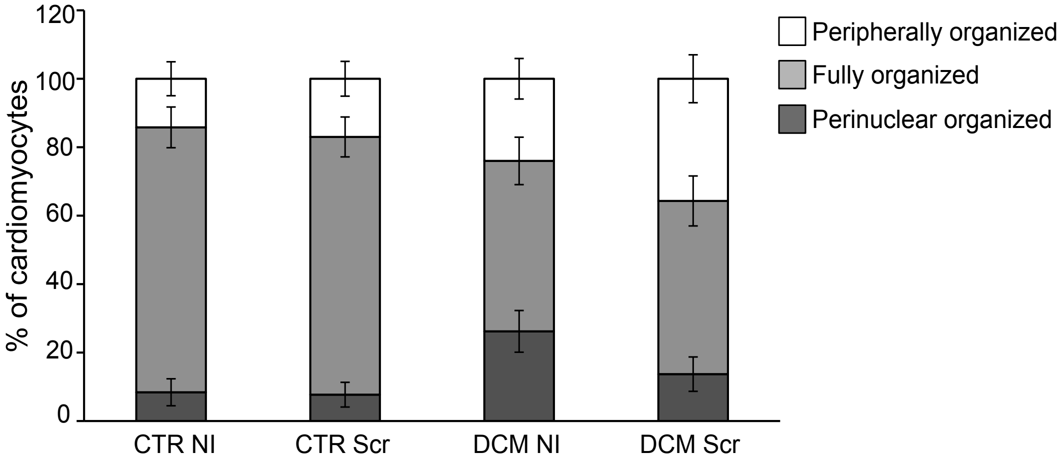

Supplementary Figure S8. Effect of lentiviral infection on sarcomere remodeling in control and DCM iPSC-derived cardiomyocytes.

The bar graph shows the percentage of perinuclear, fully and peripherally organized cells, 7 days after dissociation, in the control (CTR) and patient (DCM) groups after infection with an U7snRNA-Scramble-IRES-GFP (Scr) or in absence of infection (NI). No significant differences were observed when comparing Scr *vs* NI within the same group.

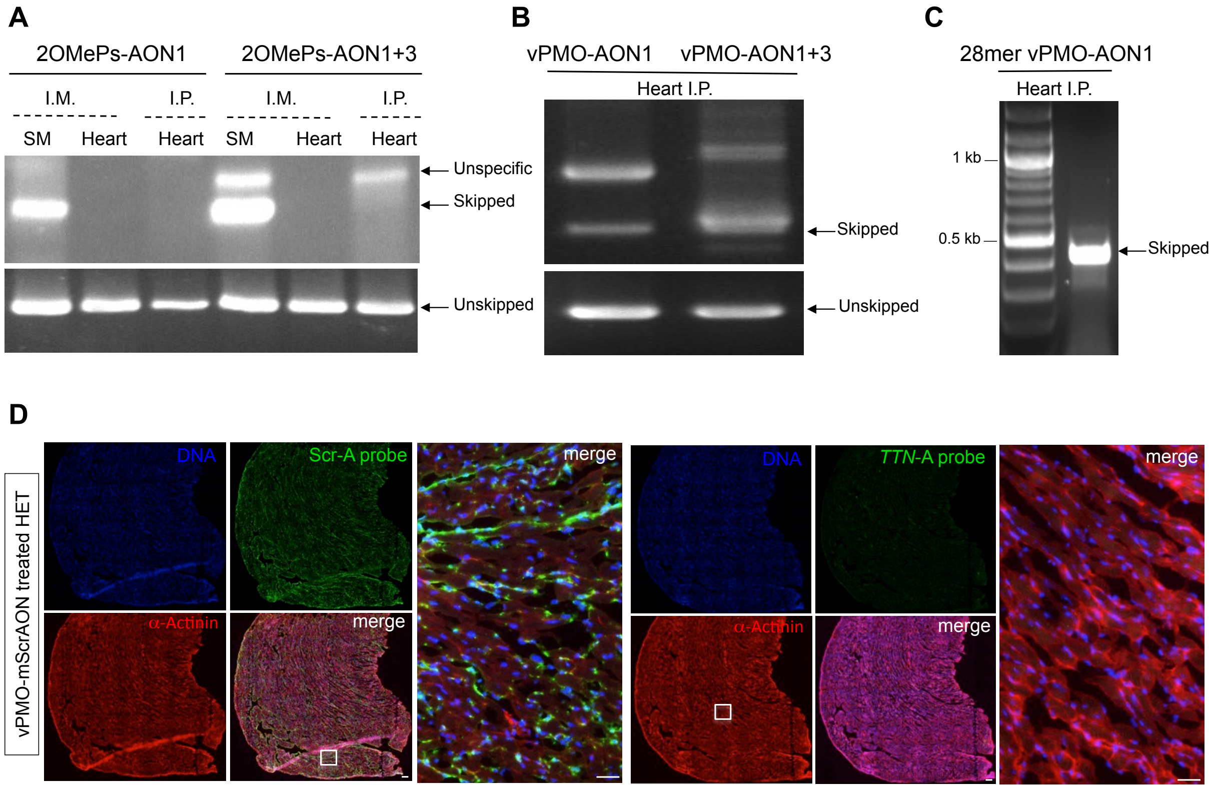

Supplementary Figure S9. ***In vivo*** **evaluation of 2OMePS and vivo-morpholino (vPMO) modified antisense oligonucleotides**.

A, RT-PCR analysis of *Ttn* exon 326 transcripts of skeletal and cardiac muscle tissue from adult knock-in mice after intramuscular (I.M., 50μg) and intraperitoneal (I.P., 6mg/kg body weight) injection of 2OMePS-AON1 and 2OMePS-AON1+3. **B,** RT-PCR analysis of *Ttn* exon 326 transcripts of cardiac muscle tissue from adult knock-in mice after I.P. injection of 23mer vPMO-AON1 and vPMO-AON1+3. **C**, RT-PCR analysis of *Ttn* exon 326 transcripts of cardiac muscle tissue from adult knock-in mice after I.P. injection of 28mer vPMO-AON1. **D**, Fluorescence in situ hybridization (FISH) of heart muscle tissue from adult knock-in mice I.P. injected with vPMO-mScrAON using probes complementary to vPMO-m*Scr*AON (left) or to vPMO-m*Ttn*AON (right). Heart sections were counterstained with sarcomeric α-Actinin. Scale bars, 250μm and 50μm (magnification).

Supplementary References

Ahuja P, Perriard E, Perriard JC, Ehler E (2004) Sequential myofibrillar breakdown accompanies mitotic division of mammalian cardiomyocytes. J Cell Sci 117: 3295-3306

Atherton BT, Meyer DM, Simpson DG (1986) Assembly and remodelling of myofibrils and intercalated discs in cultured neonatal rat heart cells. *J Cell Sci* 86: 233-248

Cox J, Mann M (2008) MaxQuant enables high peptide identification rates, individualized p.p.b.-range mass accuracies and proteome-wide protein quantification. *Nat Biotechnol* 26: 1367-1372

Cox J, Neuhauser N, Michalski A, Scheltema RA, Olsen JV, Mann M (2011) Andromeda: a peptide search engine integrated into the MaxQuant environment. *J Proteome Res* 10: 1794-1805

Goyenvalle A (2012) Engineering U7snRNA gene to reframe transcripts. *Methods Mol Biol* 867: 259-271

Goyenvalle A, Babbs A, van Ommen GJ, Garcia L, Davies KE (2009) Enhanced exon-skipping induced by U7 snRNA carrying a splicing silencer sequence: Promising tool for DMD therapy. Molecular therapy : the journal of the American Society of Gene Therapy 17: 1234-1240

Kulak NA, Pichler G, Paron I, Nagaraj N, Mann M (2014) Minimal, encapsulated proteomic-sample processing applied to copy-number estimation in eukaryotic cells. *Nat Methods* 11: 319-324

Michalski A, Damoc E, Lange O, Denisov E, Nolting D, Muller M, Viner R, Schwartz J, Remes P, Belford M, Dunyach JJ, Cox J, Horning S, Mann M, Makarov A (2012) Ultra high resolution linear ion trap Orbitrap mass spectrometer (Orbitrap Elite) facilitates top down LC MS/MS and versatile peptide fragmentation modes. Molecular & cellular proteomics : MCP 11: O111 013698

Schaab C, Geiger T, Stoehr G, Cox J, Mann M (2012) Analysis of high accuracy, quantitative proteomics data in the MaxQB database. Molecular & cellular proteomics : MCP 11: M111 014068

Sparrow JC, Schock F (2009) The initial steps of myofibril assembly: integrins pave the way. *Nat Rev Mol Cell Biol* 10: 293-298

Turnacioglu KK, Mittal B, Dabiri GA, Sanger JM, Sanger JW (1997) An N-terminal fragment of titin coupled to green fluorescent protein localizes to the Z-bands in living muscle cells: overexpression leads to myofibril disassembly. *Mol Biol Cell* 8: 705-717
