# Supplementary material for: Antisense-mediated exon skipping: a therapeutic strategy for titin-based dilated cardiomyopathy
Source: EMBO Mol Med. 2015 Mar 10;7(5):562–76. doi: 10.15252/emmm.201505047 (PMC4492817; doi:10.15252/emmm.201505047)
Supplement: Supplementary file 2 [file emmm0007-0562-sd2.pdf]

EMBO Molecular Medicine

Antisense-mediated Exon Skipping: a Therapeutic Strategy for Titin-based Dilated Cardiomyopathy

Michael Gramlich, Luna Simona Pane, Qifeng Zhou, Zhifen Chen, Marta Murgia, Sonja Schötterl, Alexander Goedel, Katja Metzger, Thomas Brade, Elvira Parrotta, Martin Schaller, Brenda Gerull, Ludwig Thierfelder, Annemieke Aartsma-Rus, Siegfried Labeit, John J. Atherton, Julie McGaughran, Richard P. Harvey, Daniel Sinnecker, Matthias Mann, Karl-Ludwig Laugwitz, Meinrad Paul Gawaz, and Alessandra Moretti

Corresponding authors: Michael Gramlich, Eberhard Karls University, Tübingen and Alessandra Moretti, Technische Universität München

---

Review timeline: Submission date: 16 January 2015
Accepted: 17 February 2015

Editor: Céline Carret

Transaction Report:

Please note that the manuscript was previously reviewed at another journal and the reports were taken into account in the decision making process at EMBO Molecular Medicine. Since the original reviews are not subject to EMBO's transparent review process policy, the reports and author response cannot be published.
